# Associations of substance use, psychosis, and mortality among people living in precarious housing or homelessness: A longitudinal, community-based study in Vancouver, Canada

**DOI:** 10.1371/journal.pmed.1003172

**Published:** 2020-07-06

**Authors:** Andrea A. Jones, Kristina M. Gicas, Sam Seyedin, Taylor S. Willi, Olga Leonova, Fidel Vila-Rodriguez, Ric M. Procyshyn, Geoffrey N. Smith, Toby A. Schmitt, A. Talia Vertinsky, Tari Buchanan, Alex Rauscher, Donna J. Lang, G. William MacEwan, Viviane D. Lima, Julio S. G. Montaner, William J. Panenka, Alasdair M. Barr, Allen E. Thornton, William G. Honer

**Affiliations:** 1 Department of Psychiatry, University of British Columbia, Vancouver, British Columbia, Canada; 2 Department of Radiology, University of British Columbia, Vancouver, British Columbia, Canada; 3 Department of Pediatrics, University of British Columbia, Vancouver, British Columbia, Canada; 4 Department of Medicine, University of British Columbia, Vancouver, British Columbia, Canada; 5 Department of Anesthesia, Pharmacology and Therapeutics, University of British Columbia, Vancouver, British Columbia, Canada; 6 Department of Psychology, Simon Fraser University, Burnaby, British Columbia, Canada; Massachusetts General Hospital, UNITED STATES

## Abstract

**Background:**

The “trimorbidity” of substance use disorder and mental and physical illness is associated with living in precarious housing or homelessness. The extent to which substance use increases risk of psychosis and both contribute to mortality needs investigation in longitudinal studies.

**Methods and findings:**

A community-based sample of 437 adults (330 men, mean [SD] age 40.6 [11.2] years) living in Vancouver, Canada, completed baseline assessments between November 2008 and October 2015. Follow-up was monthly for a median 6.3 years (interquartile range 3.1–8.6). Use of tobacco, alcohol, cannabis, cocaine, methamphetamine, and opioids was assessed by interview and urine drug screen; severity of psychosis was also assessed. Mortality (up to November 15, 2018) was assessed from coroner’s reports and hospital records. Using data from monthly visits (mean 9.8, SD 3.6) over the first year after study entry, mixed-effects logistic regression analysis examined relationships between risk factors and psychotic features. A past history of psychotic disorder was common (60.9%). Nonprescribed substance use included tobacco (89.0%), alcohol (77.5%), cocaine (73.2%), cannabis (72.8%), opioids (51.0%), and methamphetamine (46.5%). During the same year, 79.3% of participants reported psychotic features at least once. Greater risk was associated with number of days using methamphetamine (adjusted odds ratio [aOR] 1.14, 95% confidence interval [CI] 1.05–1.24, *p* = 0.001), alcohol (aOR 1.09, 95% CI 1.01–1.18, *p* = 0.04), and cannabis (aOR 1.08, 95% CI 1.02–1.14, *p* = 0.008), adjusted for demographic factors and history of past psychotic disorder. Greater exposure to concurrent month trauma was associated with increased odds of psychosis (adjusted model aOR 1.54, 95% CI 1.19–2.00, *p* = 0.001). There was no evidence for interactions or reverse associations between psychotic features and time-varying risk factors. During 2,481 total person years of observation, 79 participants died (18.1%). Causes of death were physical illness (40.5%), accidental overdose (35.4%), trauma (5.1%), suicide (1.3%), and unknown (17.7%). A multivariable Cox proportional hazard model indicated baseline alcohol dependence (adjusted hazard ratio [aHR] 1.83, 95% CI 1.09–3.07, *p* = 0.02), and evidence of hepatic fibrosis (aHR 1.81, 95% CI 1.08–3.03, *p* = 0.02) were risk factors for mortality. Among those under age 55 years, a history of a psychotic disorder was a risk factor for mortality (aHR 2.38, 95% CI 1.03–5.51, *p* = 0.04, adjusted for alcohol dependence at baseline, human immunodeficiency virus [HIV], and hepatic fibrosis). The primary study limitation concerns generalizability: conclusions from a community-based, diagnostically heterogeneous sample may not apply to specific diagnostic groups in a clinical setting. Because one-third of participants grew up in foster care or were adopted, useful family history information was not obtainable.

**Conclusions:**

In this study, we found methamphetamine, alcohol, and cannabis use were associated with higher risk for psychotic features, as were a past history of psychotic disorder, and experiencing traumatic events. We found that alcohol dependence, hepatic fibrosis, and, only among participants <55 years of age, history of a psychotic disorder were associated with greater risk for mortality. Modifiable risk factors in people living in precarious housing or homelessness can be a focus for interventions.

## Abstract 2

Andrea Jones and colleagues investigate risk factors for psychosis and mortality in unstably housed people.

## Author summary

**Why was this study done?:**

- People living in precarious housing conditions or with homelessness are at risk of poor health with complex risk factors, including substance use, mental disorders, and physical illness related to viral infection.
- Psychosis is an impairment of brain function that, to a varying extent, disconnects people from reality through hallucinations, delusions, and impaired thought processes and is associated with early mortality.
- The relative contributions of different complex risk factors to psychosis in precariously housed people are uncertain and are potentially modifiable, as is psychosis itself.

**What did the researchers do and find?:**

- We worked with 437 participants living in a neighborhood in Vancouver, Canada, meeting with each person monthly for on average 6 years, to learn how substance use and infection with HIV or hepatitis C contribute to risk for psychosis and how all these risk factors could contribute to early mortality.
- The greatest risk factor for psychosis during the study follow-up was a history of a psychotic disorder (such as schizophrenia) in the past; however, taking this into account, ongoing use of alcohol, methamphetamine, and cannabis, as well as exposure to trauma, contributed additional risk for psychosis.
- Alcohol dependence and liver damage contribute risk for early mortality; in participants under age 55, a history of a psychotic disorder contributed additional risk.

**What do these findings mean?:**

- The health challenges facing people living in precarious housing or homelessness are likely to require integrated care across substance use and mental and physical domains of health and illness.
- Targeting alcohol, methamphetamine, and cannabis use may decrease the risk for psychosis.
- Treating psychosis directly and modifying related risk factors comprehensively could contribute to decreasing high rates of early mortality.

## Introduction

People living in precarious housing or homelessness experience a “trimorbidity” of substance use disorder and mental and physical illnesses that contributes to a higher than expected mortality [1–5]. Associations between substance use and mental illness have a long history, and recent epidemiological studies support the suggestion that acute episodes of substance-induced psychosis can convert into more chronic psychotic disorders such as schizophrenia [6,7]. Psychosis, defined as a mental state with grossly impaired reality testing, operationally manifests as hallucinations and delusions [8]. Changes over time in the severity of the cardinal symptoms of psychosis (called psychotic features and rated through direct clinical interview) can be used to define a change in mental state from nonpsychotic to psychotic or vice versa [9–13]. Psychosis may be relatively persistent in people with psychotic disorders, such as schizophrenia or schizoaffective disorder, or transient, as is common in substance-related psychosis [14].

Multiple types of risk factors are associated with psychotic disorders, several of which may be present in homeless or marginally housed people. Acute psychosis may be induced by nonprescription substance use such as methamphetamine, cocaine, cannabis, and alcohol [15–18]. Independent, dose-related effects of methamphetamine and cannabis use were identified in 2 studies of individuals with psychostimulant dependence, underscoring the importance of considering their combined effects [19,20]. In addition to considering the effects of substance use, multiple biopsychosocial risk factors appear to accumulate across the life span to increase the likelihood of psychosis and associated psychotic disorders, and several of these are common in socially marginalized populations [21,22]. Adults living in precarious housing endure significant socioeconomic inequities and may have complex histories of early-life trauma and head injury [23–26]. Accumulating evidence indicates that early-life traumatic events increase risk for persistent, clinically relevant psychotic symptoms later in life [27]. Proximal traumatic events may also exacerbate risk, and together, these risk factors may have additive or synergistic effects on expression of psychosis in adulthood [28,29]. Among precariously housed individuals, traumatic brain injury (TBI) is reported in a meta-analysis to have a prevalence of over 50%, with over 20% being of at least moderate severity [25,26,30]. TBI is associated with paranoid delusions and hallucinations, even years after the injury [31].

The presence of psychotic features has relevance for health, as well as diagnosis of psychotic disorders. A study of an urban general medicine practice reported psychotic features were present in 20.9% of patients, with adverse consequences for work and social function [32]. Psychotic features are part of the diagnostic criteria for psychotic disorders, with the latter estimated to be present in 10.2%–47.2% of people living in precarious housing or homelessness [33,34]. Psychotic disorders in the general population increase the risk of mortality, particularly related to the effects of alcohol and other drugs [35–37]. A similar effect of psychotic disorders is reported for mortality of younger and middle-aged persons living in precarious housing or homelessness [38].

Comprehensive, prospective, community-based studies of risk factors for psychotic symptoms over time are scarce, and the present study was designed to create an evidence base to permit analyses of the effects of multimorbidity on health in a homeless or precariously housed sample in Vancouver, Canada [34,35]. The first objective of the present analyses was to investigate how risk exposures collectively contribute to psychotic symptoms among adults in precarious housing. Monthly data from the first year after entering a longitudinal study were analyzed to determine the effects of substance use, early-life and recent traumatic events, and past TBI on the likelihood of psychosis [23,34]. Dose–response relationships for the effects of substances and of trauma were evaluated. Time-lagged effects and possible reverse associations (psychotic features preceding increased substance use or trauma) were tested. The second objective was to examine the role of substance use, selected medical illnesses, and psychotic disorders as potential risk factors for early mortality in homeless or marginally housed persons.

## Methods

### Participants

The Hotel Study is planned as a 20-year, naturalistic, longitudinal prospective study following an adult, community-based sample [23,34,39]. Briefly, participants were recruited from single-room occupancy (SRO) hotels (n = 372) and a sequential series from the community court (n = 65) in a circumscribed neighborhood of downtown Vancouver, Canada from November 1, 2008 to October 26, 2015. All tenants in a building were potentially eligible to participate; the only criteria were being 18 years of age or older, able to communicate in English, and able to provide informed consent. The neighborhood houses approximately 3,800 residents in SRO hotels; the court processes 2,500 cases per year. Participants were homeless or lived in precarious or marginal housing, defined as being below Canadian standards for adequacy (need for repairs), affordability (rental costs <30% of before-tax income), or suitability (makeup of bedrooms and household) [40]. Rooms in SRO hotels are generally 8–12 m^2^ in size, with a sink and sometimes a hotplate. Toilet and shower facilities are shared between 10 and 15 tenants on each floor.

### Ethics statement

All participants provided written informed consent once procedures were completely described. Consent was reaffirmed at each visit thereafter. Clinically significant laboratory findings were shared with participants and their physicians. The study and the amount of an honorarium for participation were approved by the Clinical Research Ethics Boards of the University of British Columbia and Simon Fraser University.

### Baseline assessment, psychiatric diagnosis, and mortality

Sociodemographic variables were recorded by a research assistant. A psychiatrist performed a semistructured interview, a mental status examination, and a focused neurological exam. A research assistant carried out a Mini-International Neuropsychiatric Interview [41]. Healthcare records for participants’ reports of past hospitalizations for mental illness were obtained from across Canada (available from >50 years ago in some cases). Baseline psychiatric symptoms were assessed using the 30-item Positive and Negative Syndrome Scale (PANSS) [42]; functioning was assessed with the Social and Occupational Functioning Assessment Scale (SOFAS) and the Role Functioning Scale (RFS) [8,43]. Diagnoses of current and past mental and substance use disorders were made by study psychiatrists (WGH, OL, FVR) using all available information noted above, according to criteria from the Diagnostic and Statistical Manual for Mental Disorders-TR Fourth Edition (DSM-IV-TR) [8]. Coroner’s reports and records from hospitalizations for medical (nonpsychiatric) reasons were obtained for participants who died during the study period.

Screening laboratory tests were carried out at baseline and annually thereafter. These included a complete blood count and differential, liver function tests, and serology for hepatitis C virus (HCV) and human immunodeficiency virus (HIV). The aspartate aminotransferase-to-platelet ratio index (APRI) was used as a surrogate measure for hepatic fibrosis (values > 0.7) (42). More detailed descriptions of annual and monthly assessments, with references, appear in open-source publications and associated supplements [23,34].

### Assessment of time-invariant risk factors for psychotic features

**Fig 1:**
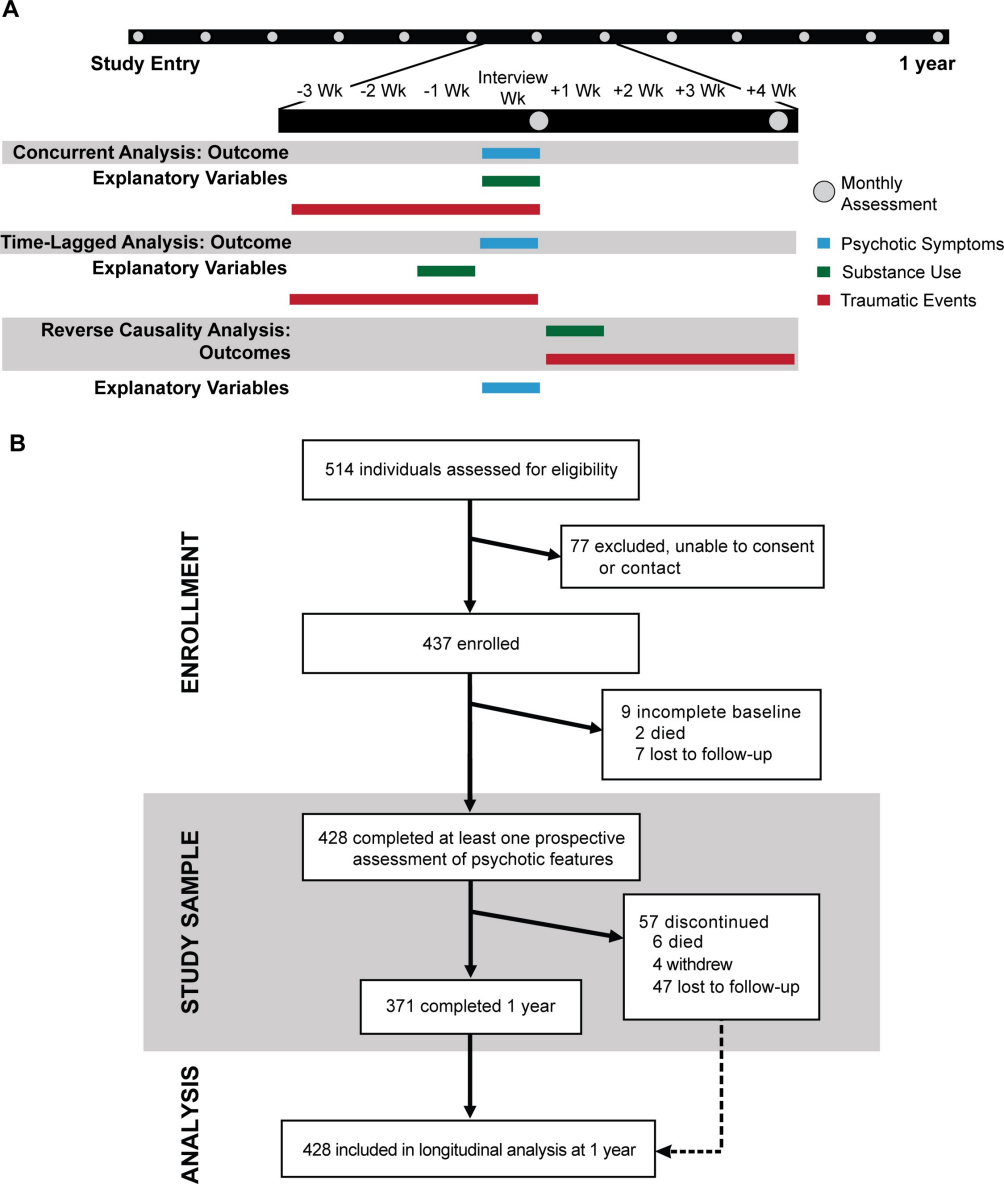
**Schematic of study design (A) and flow chart of participants (B) over the first year after study entry.** Panel A depicts the study design to evaluate time-varying factors at monthly assessments over the first year after entering the study. At each monthly assessment visit (gray circle), psychosis in the past 7 days (“Interview week” in the Fig) is evaluated by a 5-item PANSS (blue bars). Substance use is evaluated week by week over the concurrent month using the Drug Timeline Follow-back approach (green bars). Traumatic events in the concurrent month are evaluated by the THQ (red bars). Panel B depicts participant flow in the enrollment, study samples, and data analysis for the psychotic features objective. Data analysis for the mortality objective incorporated outcomes for all participants from the study beginning (November 1, 2008) to the end of the mortality analytic period (November 15, 2018). PANSS, Positive and Negative Syndrome Scale; THQ, Trauma History Questionnaire.

A comprehensive baseline assessment included standardized measures of time-invariant and time-varying risk factors (Fig 1A).

Time-invariant risk factors are those that occurred in the past and/or are not modifiable. These include past psychotic disorder diagnosis (as above), persistent sequelae from past TBI, exposure to early-life traumatic events (any, and number), and homelessness by age 18. A history of TBI was determined by clinical interview and/or high-field (3-Tesla) magnetic resonance imaging (MRI) findings identified by a neuroradiologist. Criteria for the presence of a past TBI were reporting a serious head injury with clinical manifestations (loss of consciousness ≥5 minutes or confusion ≥1 day and persistent sequelae defined as any of seizures in the past year attributable to TBI, need for seizure prophylaxis, or persistent neurological findings attributable to TBI) as used previously [23]. Past traumatic events were assessed using the semistructured Trauma History Questionnaire (THQ) [44]. Events involved objective threat of death or serious injury to which an individual reacted with extreme fear, horror, or helplessness according to the DSM-IV-TR criteria. The THQ is scored as the number of types of traumatic events endorsed to a maximum of 23, including physical, sexual, disaster-related, or crime-related events [45]. The number of types of traumatic events prior to age 18 (THQ18) was also recorded. Homelessness was defined as living on the street, in a shelter, couch surfing, or having no fixed address.

### Assessment of substance use and other time-varying risk factors for psychotic features

Monthly follow-up visits were carried out in the neighborhood study office or by outreach to the participants’ dwellings. Exposures to time-varying risk factors occurring over the concurrent and previous 3 weeks (i.e., total 1 month) were assessed, including nonprescribed substance use (types and frequency), experience of traumatic events, homelessness, and the potentially protective effect of prescription drug treatment. Nonprescription substance use and prescribed drug treatment were reported week by week using the Timeline Follow-back method [46]. Nonprescription substance use in the psychosis assessment week (i.e., the 7 days prior to each monthly visit) was examined to capture acute effects of substance exposure on symptom severity; substance use in the week prior to the assessment week was examined for persistent effects of exposure. Methamphetamine, cocaine, cannabis, opioid, alcohol, and tobacco use were reported (and confirmed by urine testing at the visit for methamphetamine, cocaine, cannabis, and opioids, n = 3,267 observations, percent agreement ranging from 83.0% to 87.1%, and kappa ranging from 0.62 to 0.68, *p* < 0.001). Days of use and route of administration were reported. Tobacco use was dichotomized (nondaily or daily use) because of the high prevalence (89.0%) among participants. Antipsychotic treatment (medication type, dose, route of administration, days of use) was reported week by week. The validity of self-reported antipsychotic use was confirmed using PharmaNet, a province-wide network that links all pharmacy dispensing records to a central database (n = 67 available records for antipsychotic use, kappa = 0.71, *p* < 0.001). Adequacy of treatment for managing psychosis was determined according to the Clinical Handbook of Psychotropic Drugs guidelines [47] and reported adherence (i.e., depot or ≥80% of past 28 days taking oral medication) in consultation with a psychopharmacologist (RMP). Prescribed methadone therapy was reported and considered adequate if taken ≥80% of the past 28 days. Adequate methadone reports exhibited high concordance with urine testing (n = 3,215 observations, kappa = 0.82, *p* < 0.001).

Recent trauma was assessed at monthly visits (Fig 1A). The number of types of traumatic events that occurred in the concurrent month (rTHQ) was recorded and analyzed as an ordinal variable truncated at 2 or more, since only 3.0% had 3 or more types of events in the concurrent month. Periods of homelessness were also captured each month.

### Outcome assessment of psychotic features

The prespecified plan was to focus on 5 key positive symptoms spanning the psychotic features description in the DSM: delusions, hallucinations, conceptual disorganization (thought disorder), suspiciousness (which may be delusional), and unusual thought content (which may be delusional). Individual PANSS items are scored on a 7-point scale, with descriptive anchors for each level of severity over the previous 7 days. Items scored 1 or 2 out of 7 are respectively “within limits of normal experience” or “questionable.” Threshold severity scores were established in advance for the 5 key items to establish the presence of clinically relevant psychotic features and are based on previous work [9–11]. Threshold scores were item-specific, calibrated to the descriptors provided for rating the PANSS as follows: (1) the threshold for the delusions and hallucinations items according to PANSS descriptors is a score of 3 (mild), indicating these symptoms are definitely present, consistent with the psychotic features definition according to the DSM and categorically different from a state of good mental health. (2) The threshold for the items of conceptual disorganization and unusual thought content was set to a score of 4 (moderate). For these items, according to PANSS descriptors, a score of 3 (mild) may or may not be associated with formal thought disorder or bizarre delusions. For this reason, the threshold score was set to a higher level, consistent with the use of similar items as inclusion criteria for clinical trials of antipsychotic medications [48]. (3) The threshold for the suspiciousness item was set to an even higher threshold value of 5 (moderately severe) because only at this level or above is the descriptor provided in the PANSS consistent with a paranoid delusion [42].

We examined the inter-rater reliability of this assessment strategy. Two raters carried out separate interviews of 26 participants on the same day. The reliability for presence or absence of at least 1 of a possible 5 symptoms meeting the threshold for psychotic features was kappa = 0.69 (*p <* 0.001). This is a more stringent approach than is used in most studies, in which inter-rater reliability for individual PANSS items is reported by having different raters observe the same video of a patient interview or by having 2 raters carry out a joint interview of a patient. Of the few available studies using independent raters, and independent interviews of the same patient, a test–retest reliability intraclass correlation coefficient = 0.65 (95% confidence interval [CI] 0.62–0.68) was reported for the crosscutting domain of psychosis (hallucinations or delusions) [49].

### Statistical analysis

Descriptive statistics of participants’ time-invariant and time-varying risk factors for psychotic features were reported as mean (standard deviation) or median (interquartile range) for continuous variables and number (proportion) for categorical variables. The associations between a past history of a psychotic disorder diagnosis and current functioning at baseline were assessed using analysis of covariance, controlling for age and sex. Similar analyses were carried out with psychotic features at baseline.

The first objective was to investigate how risk exposures collectively contribute to psychotic features among adults in precarious housing. Using data from monthly visits over the first year following each participant’s study entry, mixed-effects logistic regression models with random intercept and slope for longitudinal binary outcome data were used to assess the relationships between psychotic features (presence or absence during the psychosis assessment week) and exposure to risk factors. Fixed effects estimated the main effects of risk factors on odds of psychotic features. Time-invariant risk factors included past psychotic disorder diagnosis (presence or absence), persistent sequelae from past TBI (presence or absence), homelessness by age 18 (presence or absence), and past exposure to traumatic events (dose–effect estimate). Time-varying risk factors included number of days of nonprescription substance use, exposure to recent traumatic events (dose–effect estimates), and homelessness in the concurrent month (present or absent). Linearity of dose–effect estimates was tested by modeling factors as continuous and ordinal predictors in separate models and testing both linear and quadratic effects. Other covariates included age, sex, time point, antipsychotic treatment, and methadone therapy. Random effects of subject and time were included to account for between-subject variability and within-subject correlation across repeated measures, respectively. Random effect standard deviations were reported. Adjusted models were estimated by a stepwise model fitting process iteratively adding and removing random effects, then time-invariant and time-varying fixed effects variables that were significantly associated with the outcome in bivariate analyses [50]. The best fitting multivariable model was identified by successively comparing nested models using a likelihood ratio test and comparing Akaike Information Criterion (AIC) values. Model diagnostic plots were assessed for normality of the distribution of residuals. We tested multiplicative interactions using the Wald test. Predicted probabilities for psychotic features were estimated for combinations of significant risk factors.

Several approaches were employed to test the robustness of results (Fig 1A). Time-lagged substance use during the week prior to the psychosis assessment week was included in the adjusted model to estimate the persistence of substance use effects. Reverse association (sometimes described as “reverse causality,” although causality cannot be determined) was investigated by estimating the effect of psychotic features (presence or absence) on the days of substance use in the week following the psychosis assessment week using linear mixed-effects models and on the likelihood of a traumatic event during the following month using logistic mixed-effects models, adjusting for covariates. Further, the validity of self-reported substance use was tested by substituting urine results from the psychosis assessment visit for self-report in adjusted models. Sensitivity analyses assessed for evidence of systematic bias and the pattern of missingness by comparing participants who had missing or available variables of interest, who remained engaged in the study versus who discontinued, and who were included or excluded from longitudinal analyses using chi-squared and Wilcoxon rank–sum tests. Multiple imputation analysis was performed by imputing values generated from regression models with relevant predictors, including covariates of the main model of interest and variables related to a variable’s missingness. First, we imputed 10 completed data sets with 10 iterations for each imputation analysis. Second, the main model of interest was fitted to each of the completed data sets. Last, the parameter estimates from the second step were pooled to produce final parameter estimates. Pooled parameter estimates from the imputed data sets were compared to those from complete-case analysis to test whether our findings and inferences were affected by missing data.

The second objective was to examine the role of substance use, selected medical illnesses, and psychotic disorders as potential risk factors for early mortality, focusing on previously reported risk factors in the present sample examined over a shorter time period [38,51,52]. These analyses were not prespecified. Left-truncated and right-censored Cox regression models, with age as the timescale, were used to examine the effect of psychosis (a past history of psychotic disorder and/or psychotic features present during the first year after study entry), substance use, and related physical illness (hepatic fibrosis, HIV at baseline) on the risk of mortality for the time period November 1, 2008 to November 15, 2018. Statistical tests and visual plots of the Schoenfeld residuals were used to assess the assumption of proportionality of hazards. Violations of proportionality (i.e., significant Schoenfeld residual global test) were addressed by stratifying the sample using an age change point (threshold age separating a younger and an older group), determined by visual inspection of the Schoenfeld residual-by-age plots and identification of the age corresponding to the inflection point of the smoothing spline fit to the plots. Kaplan–Meier survival curves were constructed to plot the effects of statistically significant predictors. All analyses were performed in R (version 3.4.0; R Core Team) and RStudio (version 1.0.143; R Foundation for Statistical Computing). Significance was set to alpha level of 0.05, and all *p*-values are 2-sided. Additional details on methodology appear in the S1 STROBE Checklist and the S1 Protocol.

## Results

### Demographics and clinical characteristics of participants

Staggered participant enrollment and flow appears in Fig 1B. Demographic and clinical characteristics of participants appear in Table 1.

**Table 1:** Characteristics of participants at study entry and during the first year following study entry.

At Study Entry	N = 437
Age (years)	40.6 (11.2)
Men	340 (77.8%)
Ethnicity/race	
—White	261 (59.7%)
—Indigenous	113 (25.9%)
—Other	63 (14.4%)
Completed high school or equivalent	186/433 (43.0%)
Past homelessness	323/431 (74.9%)
Receiving income assistance	422/433 (97.5%)
Past psychotic disorder diagnosis	266 (60.9%)
—Substance-induced psychosis	110 (25.2%)
—Psychosis not otherwise specified	55 (12.6%)
—Schizophrenia	40 (9.2%)
—Schizoaffective disorder	29 (6.6%)
—Depression or bipolar with psychosis	28 (6.4%)
—Other psychotic disorder*	4 (0.9%)
Past substance dependence diagnosis	
—Cocaine	320 (73.2%)
—Opioid	256 (58.6%)
—Alcohol	210 (48.1%)
—Cannabis	194 (44.4%)
—Methamphetamine	159 (36.4%)
—Other	60 (13.7%)
SOFAS score† (n = 425)	39.9 (10.6)
PANSS‡ (n = 359)	
—Total score	69.7 (17.5)
—Positive subscale	16.0 (6.0)
—Negative subscale	16.5 (5.8)
HIV seropositive	65/409 (15.9%)
Hepatic fibrosis (APRI > 0.7)	70/395 (17.7%)
**During First Year after Entering Study (Analysis Data Set)**	**N = 428**
Monthly assessment visits over first year, no. per participant	9.8 (3.6)
Frequency of participants with psychotic features**	
—Participants with psychotic features absent in all visits over first year	93 (21.7%)
—Participants with psychotic features present in 1 visit over first year	51 (11.9%)
—Participants with psychotic features present in ≥2 visits over first year	284 (66.4%)
Participants with psychotic features at ≥1 visit, according to symptom type present	**N = 335**
—Delusions	302 (90.1%)
—Conceptual disorganization	144 (43.0%)
—Hallucinatory behavior	248 (74.0%)
—Suspiciousness/persecution	131 (39.1%)
—Unusual thought content	157 (46.9%)
—Only 1 symptom type above threshold (monosymptomatic)	64 (19.1%)
—≥2 symptom types above threshold (polysymptomatic)	271 (80.9%)

Data are n/N (%), mean (SD).

*N = 1 delusional disorder, N = 3 psychosis due to a medical condition

†SOFAS is rated 0–100, with higher scores representing better functioning.

‡PANSS is a 30-item scale rated after an interview and mental status examination by a psychiatrist, used to assess the severity of positive and negative symptoms of psychosis and general mental health.

**Five key positive symptom items from the PANSS were rated at each monthly visit by a research assistant and serve as the primary data for presence of psychotic features outcome during the first year after study entry.

**Abbreviations:** APRI, aspartate aminotransferase-to-platelet ratio index; HIV, human immunodeficiency virus; PANSS, Positive and Negative Syndrome Scale; SOFAS, Social and Occupational Functioning Assessment Scale.

At baseline, 425 of 437 (97.3%) participants were living in precarious housing, and 12 (2.7%) were homeless. The demographic characteristics of participants were similar to other reports from this Vancouver neighborhood and to reports from Canadian studies of precariously housed or homeless people in other cities (S1 Table, S2 Table).

The majority of participants had experienced past psychotic disorders and substance dependence (Table 1). In 4,285 assessments carried out across all participants over the first year following study entry, with a mean (SD) of 9.8 (3.6) visits per person, 335 of 428 (78.3%) participants endorsed positive symptom severity indicating the presence of 1 or more psychotic features according to the DSM on at least 1 visit (Table 1). Considering all assessments aggregated across participants over the first year after study entry, a single psychotic symptom was recorded in 781 of 4,285 (18.2%) of assessments; 2 or more psychotic symptoms were present in 1,069 of 4,285 (24.9%) of assessments. In assessments with 2 or more psychotic symptoms present, delusions and hallucinations were the most frequent combination, occurring in 709 of 1,069 (66.3%), followed by delusions and unusual thought content in 561 of 1,069 (52.5%).

At baseline, analyses of covariance showed participants’ levels of social and occupational function and of role functioning were associated with both a past history of psychotic disorder and the presence of current psychotic features, adjusting for age and sex (S3 Table, S4 Table). In combined analyses, the associations with current psychotic features predominated (SOFAS: estimate 2.3, standard error [SE] 0.6, *p* < 0.001; RFS: estimate 0.8, SE 0.2, *p* < 0.001).

### Associations between risk factors and psychotic features

The characteristics of risk factors for psychotic features are described in Table 2.

**Table 2:** Descriptive characteristics of time-invariant and time-varying risk factors for the presence of psychotic features in visits over the first year after study entry.

Risk Factor	N = 428
**Time-invariant risk factors**	
Persistent clinical or MRI evidence of past TBI*	43/428 (10.0%)
Traumatic events by age 18 (THQ score ≥1)	330/420 (78.6%)
THQ items endorsed by age 18, no. (n = 420)	2.0 (1.0–4.0)
Homelessness by age 18	131/424 (30.9%)
**Time-varying risk factors over the first year after entering study**	
Any nonprescribed substance use over first year	
—Tobacco (any daily use)	381/428 (89.0%)
—Alcohol	331/427 (77.5%)
—Methamphetamine	198/426 (46.5%)
—Cannabis	310/426 (72.8%)
—Cocaine	312/426 (73.2%)
—Opioids	221/426 (51.9%)
Frequency of substance use over first year (users only) days per week	2.0 (1.0–3.0)
—Alcohol use†	2.0 (1.0–5.0)
—Methamphetamine use	3.0 (1.0–7.0)
—Cannabis use	7.0 (2.0–7.0)
—Cocaine use	5.0 (2.0–7.0)
—Opioid use	7.0 (2.0–7.0)
Participants experiencing any traumatic event(s) over first year (rTHQ score ≥1)‡	334/410 (81.5%)
Months over first year with traumatic events for participants with rTHQ score ≥1	
—Months with no traumatic events	7 (4–9)
—Months with 1 traumatic event	1 (0–3)
—Months with ≥2 traumatic events	0 (0–1)
Homeless ≥1 time over first year	42/428 (9.8%)
Any prescribed substance use over first year	
—Adequate antipsychotic treatment	101/427 (23.7%)
—Adequate methadone maintenance therapy	166/427 (38.9%)

Data are n/N (%) or median (interquartile range). **Abbreviations:** CT, computed tomography; MRI, magnetic resonance imaging; rTHQ, recent THQ; TBI, traumatic brain injury; THQ, Trauma History Questionnaire.

*Clinical evidence n = 19 (loss of consciousness ≥5 minutes or confusion ≥1 day AND persistent clinical symptoms such as seizures or cognitive impairment) or MRI/CT evidence (n = 24) of previous TBI.

†For alcohol, users had frequent weeks with no use, so value refers to weeks when using only.

‡rTHQ occurring in the month concurrent with the monthly psychosis assessment visit, carried out by a research assistant.

Persistent sequelae of past TBI indicated the consequences of physical trauma. Multiple types of traumatic life experiences before age 18, with potential implications for the development of mental disorders, were common. During the first year after study entry, participants used nonprescribed substances, in increasing order: methamphetamine, opioids, cannabis, cocaine, alcohol, and tobacco. In users, frequency of substance use over the first year varied from once per week to daily. Most participants experienced traumatic events at least once over the year; periods of homelessness were also noted as potential stressors. Adequate antipsychotic treatment was less common than adequate methadone treatment.

Effects of time-invariant and time-varying risk factors for psychotic features observed in the mixed-effects logistic regression models are summarized in Table 3.

**Table 3:** Effects of risk factors on odds of observing psychotic features in visits during the first year after entering the study.

	Unadjusted			Adjusted* (n = 409, 3,625 Observations)	
	n, Observations	OR (95% CI)	*p*-Value	OR (95% CI)	*p*-Value
**Covariates**					
Time (months)	428, 4,294	0.94 (0.91–0.97)	<0.001	0.96 (0.93–1.00)	0.07
Age	428, 4,294	0.96 (0.94–0.99)	0.002	1.00 (0.98–1.03)	0.78
Male	428, 4,294	1.79 (0.94–3.39)	0.07	1.82 (1.03–3.21)	0.04
**Time-invariant risk factors**					
Past psychotic disorder diagnosis	428, 4,294	14.05 (8.96–22.03)	<0.001	17.77 (10.83–29.15)	<0.001
THQ score by age 18†	420, 4,207	1.18 (1.07–1.31)	0.001	–	–
Persistent sequelae of TBI	428, 4,294	1.36 (0.56–3.29)	0.49	–	–
Homelessness by age 18	424, 4,256	1.16 (0.65–2.07)	0.62	–	–
**Time-varying risk factors over the first year after entering the study**‡					
**Concurrent week of psychosis assessment**					
Daily tobacco use	428, 4,193	1.49 (0.96–2.31)	0.07	–	–
Days using alcohol	427, 4,262	1.11 (1.03–1.20)	0.007	1.09 (1.01–1.18)	0.04
Days using methamphetamine	426, 4,233	1.13 (1.05–1.22)	0.002	1.14 (1.05–1.24)	0.001
Days using cannabis	426, 4,235	1.08 (1.03–1.13)	0.002	1.08 (1.02–1.14)	0.008
Days using cocaine	426, 4,235	1.07 (1.01–1.13)	0.009	–	–
Days using opioid	426, 4,234	1.05 (0.99–1.12)	0.08	–	–
**Concurrent month of psychosis assessment**					
rTHQ score	410, 3,775	1.50 (1.16–1.93)	0.002	1.54 (1.19–2.00)	0.001
Homelessness	428, 4,230	1.35 (0.65–2.80)	0.41	–	–
Adequate antipsychotic treatment	427, 4,158	2.78 (1.76–4.40)	<0.001	2.04 (1.26–3.30)	0.004
Adequate methadone therapy	427, 4,147	0.90 (0.62–1.30)	0.58	–	–

Data are OR and 95% CI. **Abbreviations:** AIC, Akaike Information Criterion; CI, confidence interval; OR, odds ratio; rTHQ, recent THQ; TBI, traumatic brain injury; THQ, Trauma History Questionnaire.

*Adjusted model selected for optimal fit by AIC and likelihood ratio test. Fixed effects are adjusted for the included time-invariant, time-varying factors, and covariates. Random effects (standard deviation): subject (1.94), time point (0.22).

†Linear effects of THQ scores for number of traumatic events by age 18. Quadratic effects were not significant (*p* > 0.10).

‡Linear effects of number of days of substance use in the week concurrent with the psychosis assessment visit and rTHQ scores for the number of traumatic events (0, 1, or ≥2) in the month concurrent with the assessment visit are reported. Quadratic effects were not significant (*p* > 0.10).

The most potent risk factor was a past diagnosis of a psychotic disorder (see also Table 1). The final adjusted model (Table 3) indicated independent, linear, dose-dependent effects of days of alcohol, of methamphetamine, and of cannabis use in the concurrent week on odds for the presence of psychotic features. Greater exposure to trauma in the concurrent month was linearly associated with greater odds of psychotic features. The odds of experiencing psychotic features were greatest among males. Additionally, the presence of psychotic features was associated with receiving adequate antipsychotic treatment in the past month (likely indicating at least some of those in need did access treatment, but with incomplete response). These effects did not change over time during the year, and there was no evidence of multiplicative interactions. Other putative risk factors, including homelessness in the concurrent month, were not associated with experiencing psychotic features, nor was time itself associated with risk.

### Time-lagged effects of substance use and potential reverse associations

**Fig 2:**
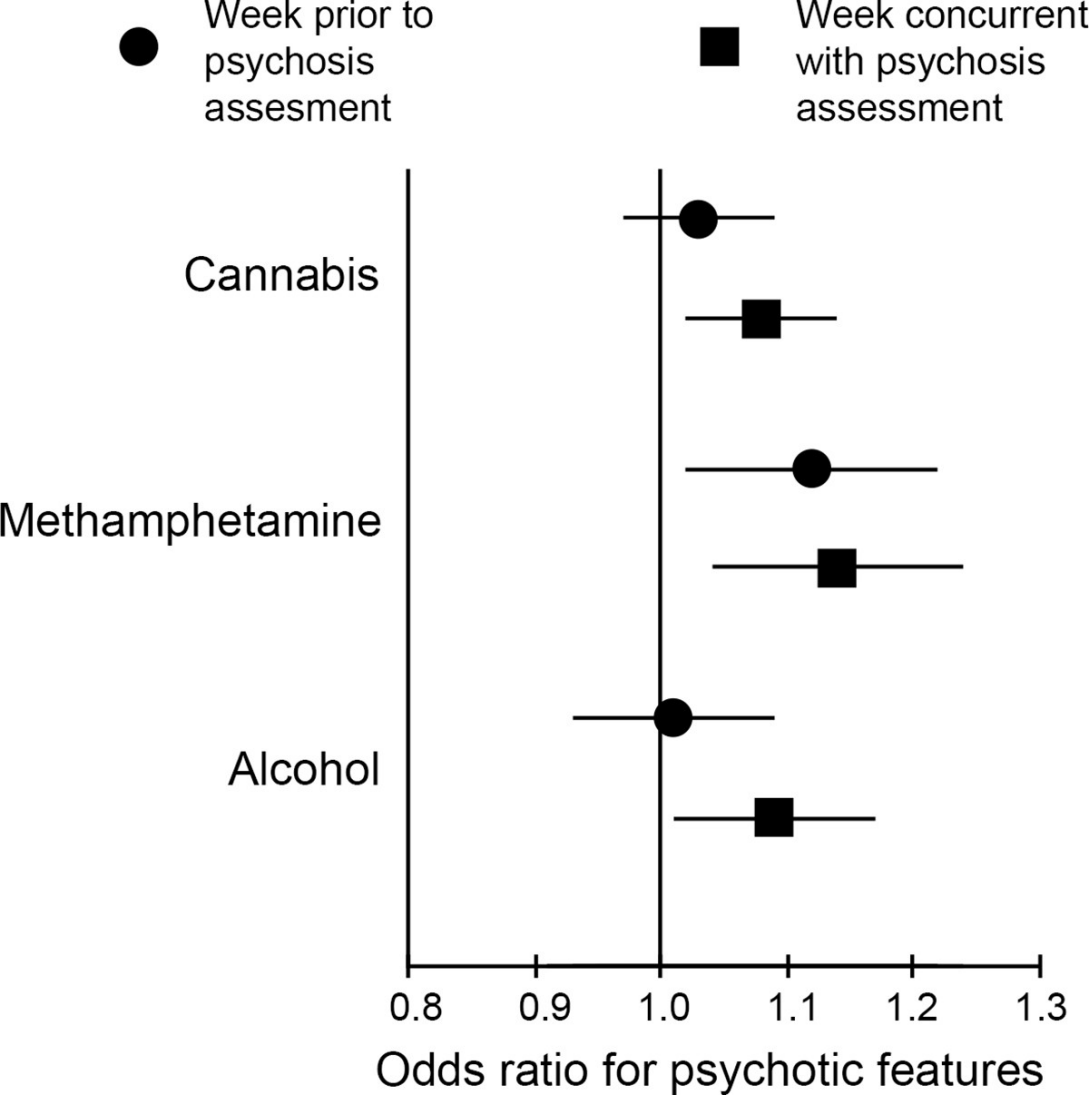
Association between psychotic features and days of use of substances in the concurrent or the prior week. In the concurrent week, use of all 3 substances increased likelihood of expression of psychotic features, suggesting acute effects; in the prior week, only use of methamphetamine was associated with increased likelihood of psychotic features in the assessment (concurrent) week.

For longer-acting substances, use in the week prior to the psychosis assessment week could contribute to the expression of psychotic features. To study this, a time-lagged analysis was carried out using the same mixed-effects logistic regression models as above, with substance use in the prior week substituted for substance use in the week concurrent with the psychosis assessment (Fig 1B and S5 Table). Time-invariant and concurrent month risk factor effects were unchanged. Use of cannabis or alcohol in the week prior did not contribute to the likelihood of psychotic features being observed in the psychosis assessment week (Fig 2).

Methamphetamine use showed a different pattern, as use in the prior week was associated with psychotic features in the psychosis assessment week (aOR = 1.12, 95% CI 1.03–1.22, *p* = 0.012). This suggests the effects of methamphetamine as a risk factor for psychotic features may be relatively longer lasting and/or the effects of cannabis and alcohol are more acute and transient. Next, the possibility of reverse association was analyzed using psychotic features as the predictor of substance use in the week following the psychosis assessment week (Fig 1B). No significant associations were observed for alcohol, methamphetamine, cannabis, or cocaine use (all *p* > 0.05, S6 Table). The presence of psychotic features at assessment was associated with the outcome of new traumatic experiences in the following month (OR = 1.27, 95% CI 1.04–1.55, *p* = 0.02). A similar relationship was observed when the covariates of time, age, sex, past psychotic disorder diagnosis, days of other nonprescription substance use in the following week, and adequate antipsychotic treatment in the following month were added to the model (OR = 1.30, 95% CI 1.05–1.62, *p* = 0.02).

### Sensitivity analyses

Effects of substance use in the psychosis assessment week on likelihood of psychotic features were similar in mixed-effects logistic regression models, substituting urine drug screen results at the visit for self-reported substance use as risk factors (Table 4).

**Table 4:** Effects of urine drug screen results rather than self-report of substance use on odds of observing psychotic features in visits during the first year after entering the study.

	Unadjusted			Adjusted* (n = 389, 2,816 Observations)	
	n, Observations	OR (95% CI)	*p*-Value	OR (95% CI)	*p*-Value
**Covariates**					
Time (months)	428, 4,294	0.94 (0.91–0.98)	<0.001	0.96 (0.92–1.00)	0.06
Age	428, 4,294	0.96 (0.94–0.99)	0.002	1.02 (0.99–1.04)	0.20
Male	428, 4,294	1.79 (0.94–3.39)	0.07	1.85 (1.05–3.27)	0.03
**Time-invariant risk factors**					
Past psychotic disorder diagnosis	428, 4,294	14.05 (8.96–22.03)	<0.001	15.04 (9.18–24.64)	<0.001
THQ score by age 18†	420, 4,207	1.18 (1.07–1.31)	0.001	–	–
Persistent sequelae of TBI	428, 4,294	1.36 (0.56–3.29)	0.49	–	–
Homelessness by age 18	424, 4,256	1.16 (0.65–2.07)	0.62	–	–
**Time-varying risk factors over the first year after entering the study**					
**Concurrent week urine drug screen**					
Methamphetamine	415, 3,276	2.05 (1.48–2.83)	<0.001	2.44 (1.72–3.44)	<0.001
Cannabis	415, 3,274	1.79 (1.33–2.42)	<0.001	1.47 (1.06–2.02)	0.02
Cocaine	415, 3,277	0.88 (0.64–1.21)	0.43	–	–
Opioid	415, 3,273	1.27 (0.95–1.71)	0.11	–	–
**Concurrent month**					
rTHQ score‡	410, 3,775	1.50 (1.16–1.93)	0.002	1.52 (1.14–2.03)	0.004
Homelessness	428, 4,230	1.35 (0.65–2.80)	0.41	–	–
Adequate antipsychotic treatment	427, 4,158	2.78 (1.76–4.40)	<0.001	1.91 (1.13–3.23)	0.02
Adequate methadone therapy	427, 4,147	0.90 (0.63–1.30)	0.58	–	–

Data are OR and 95% CI. **Abbreviations:** CI, confidence interval; OR, odds ratio; rTHQ, recent THQ; TBI, traumatic brain injury; THQ, Trauma History Questionnaire.

*Adjusted for all time-invariant, time-varying factors, and covariates included. Random effects (standard deviation): subject (1.71), time point (0.22).

†Linear effects of THQ scores for number of traumatic events by age 18. Quadratic effects were not significant (*p* > 0.10).

‡Linear effects of rTHQ scores for the number of traumatic events (0, 1, or ≥2) in the month concurrent with the assessment visit are reported. Quadratic effects were not significant (*p* > 0.10).

Sensitivity analyses revealed no evidence of systematic bias in the pattern of missing data. Participants included in longitudinal analyses did not differ from excluded individuals, except they were older (chi-squared [1] = 6.67, *p* = 0.01). Participants with missing psychosis assessments were younger (estimate = −0.034, SE = 0.013, *p* = 0.007), and otherwise, missingness was not associated with psychotic features or risk factors. Participants who discontinued (e.g., moving from city, incarceration, living in treatment facility, lost contact) had greater THQ18 scores (Wilcoxon = 16,367, *p* = 0.039) but were otherwise similar on all factors to those who remained in the study. Altogether, data were determined to be missing at random, with missingness unrelated to psychosis or related factors, and we proceeded with the multiple imputation procedure to test whether missingness impacted the findings. Death, psychosis, and psychosis risk factors were included as potentially relevant predictors of missingness in the imputation procedure. Pooled parameter estimates from imputed data sets were similar to complete-case analysis results (S7 Table), suggesting that the present findings were not affected by missing data.

### Psychosis and risk of early mortality

Participants continued with monthly follow-ups after the first year in the study, and as of November 15, 2018, participants were followed for a median of 6.3 years (25th–75th percentiles, 3.1–8.6 years) after study entry. During 2,481 person years of observation, 79 of 437 (18.1%) participants died. Causes of death were physical illness (40.5%), accidental overdose (35.4%), trauma (5.1%), suicide (1.3%), and unknown (17.7%). Unadjusted Cox proportional hazards regression analyses on the full sample indicated associations between mortality risk and evidence of hepatic fibrosis, HIV seropositive status, and baseline (but not past) alcohol dependence (Table 1 and S8 Table). Risks associated with past or baseline dependence on cocaine, methamphetamine, opioids, cannabis, or daily cigarette smoking were not statistically significant, nor was HCV-seropositive or qualitative polymerase chain reaction (qPCR)-positive status. Adjusted Cox regression analysis indicated statistically significant risks associated with hepatic fibrosis and alcohol dependence at baseline (Table 5).

**Table 5:** Adjusted survival analysis. The effect of past history of psychotic disorder on mortality interacted with age; results are presented for 2 age groupings.

	Model: Whole Sample (n = 391)		Model: Age <55 Years (n = 281)		Model: Age ≥55 Years (n = 110)	
Risk Factor	HR (95% CI)	Log-Rank *p*-Value	HR (95% CI)	Log-Rank *p*-Value	HR (95% CI)	Log-Rank *p*-Value
Baseline alcohol dependence	1.83 (1.09–3.07)	0.02	1.85 (0.89–3.87)	0.10	1.55 (0.72–3.37)	0.27
Hepatic fibrosis (APRI > 0.7)	1.81 (1.08–3.03)	0.02	1.76 (0.76–4.08)	0.19	1.55 (0.76–3.17)	0.23
HIV positive	1.69 (0.99–2.87)	0.06	0.93 (0.38–2.28)	0.87	1.92 (0.92–4.00)	0.08
Past history psychosis			2.38 (1.03–5.51)	0.04	0.76 (0.39–1.48)	0.43

Results are HR and 95% CI. **Abbreviations:** APRI, aspartate aminotransferase-to-platelet ratio index; CI, confidence interval; HIV, human immunodeficiency virus; HR, hazard ratio.

The effects on mortality of both a past history of psychotic disorder and psychotic features occurring during the first year after entering the study interacted with age as determined by a significant Schoenfeld residual global test (past history *p* = 0.04, first year psychotic features *p* = 0.02, S8 Table). By visual inspection of Schoenfeld residual plots, the change-point age separating younger and older groups was set at 55 years, consistent with earlier reports [38,52]. Unadjusted Cox proportional hazards regression analyses on the group less than 55 years of age showed an association between past history of psychosis and early mortality; no association was observed for psychotic features in the year following study entry, nor were associations observed in the older group (S8 Table). In those less than 55 years of age, a past history of psychotic disorder was associated with early mortality in a model controlled for baseline alcohol dependence, hepatic fibrosis, and HIV status (Table 5).

**Fig 3:**
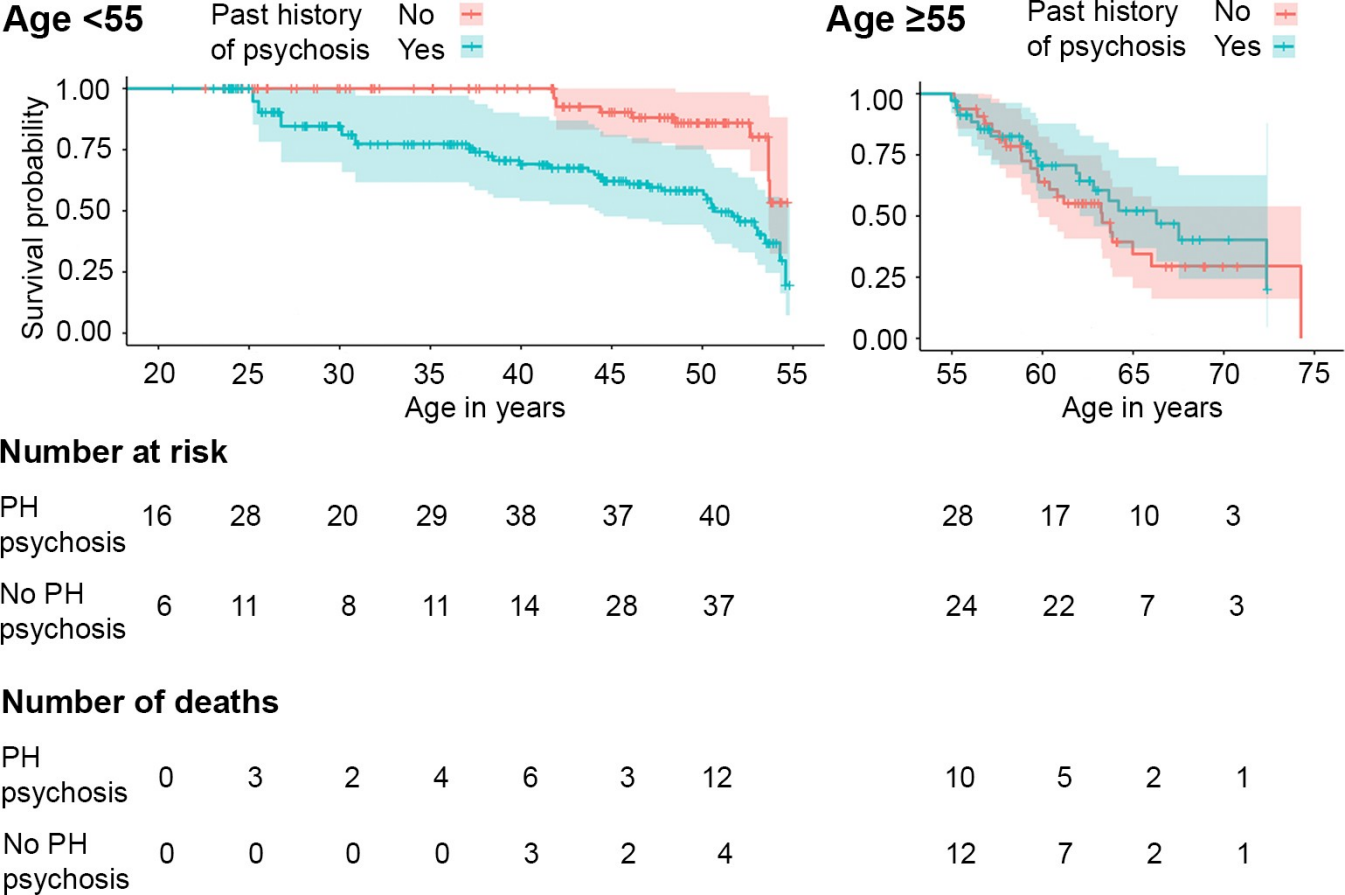
Kaplan–Meier curves for the probability of survival by age among residents of precarious housing. Left panel <55 years old, right panel ≥55 years old, comparing those with and without a history of a psychotic disorder. PH psychosis, past history of a psychotic disorder.

There were 39 deaths among 323 (12.1%) in the younger group and 40 among 114 (35.1%) in the older group. The distribution of causes of death did not differ between the younger and older groups or between those with or without a past history of psychotic disorder. As seen in Fig 3, for participants in the younger group, a past history of psychotic disorder (208 of 323, 64.4%) was associated with earlier death (30 of 208, 14.4% versus 9 of 115, 7.8%). In contrast, for participants age 55 or older, a past history of psychotic disorder (58 of 114, 50.9%) did not increase risk for early mortality (18 of 58, 31.0% versus 22 of 56, 39.3%).

## Discussion

In this sample of people living in precarious housing or homelessness in an impoverished neighborhood of Vancouver, Canada, we found that psychotic features were prominent and were associated with functional impairment. Not surprisingly, the most potent risk factor was a diagnosis of past history of a psychotic disorder. In adjusted analyses, methamphetamine, alcohol, and cannabis use also each showed associations with psychotic features; tobacco, cocaine, and opioids did not. Experiencing recent trauma also increased risk. In adjusted analyses, alcohol dependence and hepatic fibrosis both contributed to increased mortality; risk related to HIV was increased, but not statistically significant, in the adjusted analysis. The effects of psychotic disorders and psychotic features on mortality risk differed according to age. For those less than 55 years old, a past psychotic disorder increased mortality risk, adjusting for risks related to alcohol dependence, hepatic fibrosis, and HIV. The effect of past psychotic disorder was not seen for older study participants.

One of the earliest accounts of amphetamine-related psychosis described a group of patients who developed psychosis after a single dose of amphetamine or methamphetamine and another group in which the onset of psychotic features was after periods of days to years of use [53]. We observed effects related to days of use in the concurrent week and in the week prior. This finding is consistent with an earlier Hotel Study report relating the severity of positive symptoms of psychosis to the number of days using during the full month before assessment in a subsample of methamphetamine-dependent participants [20]. Similar dose and duration effects for methamphetamine in dependent users are reported by others [19,54,55].

Descriptions of the role of alcohol as a risk factor or precipitant for psychotic features span 3 centuries [56,57]. An acute onset is described, consistent with the present finding of increased odds of psychotic features in weeks concurrent with alcohol use but, in contrast to methamphetamine, less carry-over effect from use in the week prior. This could relate to the faster metabolism of alcohol compared with methamphetamine but could also represent a difference in the nature of the symptoms. Psychotic features related to methamphetamine may persist longer than 1 month in as many as 25% of users [58]. In recent epidemiological studies, alcohol-induced psychosis was the most frequent type of substance-induced psychosis yet was least likely to be associated with a later diagnosis of schizophrenia [6,7]. However, alcohol-induced psychotic disorder was reported to be associated with an increased risk of mortality [59].

The present findings of an association between recent cannabis use and psychotic features are consistent with reports of cannabis creating an acute toxic psychosis and with short-duration effects in laboratory studies of high doses of intravenous tetrahydrocannabinol (THC) in 35%–50% of healthy participants [17,60,61]. The concentration of THC and of the potentially antipsychotic cannabidiol in the cannabis used by Hotel Study participants is unknown. In contrast to alcohol-related psychosis, in epidemiological studies, cannabis- and methamphetamine-induced psychotic disorders were more likely to be associated with progression to schizophrenia or other persistent psychotic disorder [6,7]. Larger samples of more homogenous groups of participants would be needed to address more specific questions related to the mechanism of action of cannabis to increase risk for persistent psychotic features and the potentially moderating effects of psychotic disorders. The present results do suggest that in a relatively large, heterogeneous group of people living in precarious housing, cannabis use is not without risk in contributing to psychotic features, even when concurrent substance use and other risk factors are included in the model. There was no evidence for a temporal association between psychotic features as a risk factor for subsequent increased substance use.

Cocaine use in the present sample was not associated with increased risk for psychotic features. A clinical account at the time of a widespread increase in use of crack cocaine reported prominent psychotic features, and a laboratory study administering intravenous cocaine in a binge-like schedule observed symptoms of paranoia [62,63]. In a previous report from the Hotel Study, psychotic features were less prominent in cocaine-dependent than in methamphetamine-dependent participants [64]. Differences in patterns of use may contribute to these findings, as may differences in the mechanism of action of cocaine and methamphetamine [54].

The present analysis did not demonstrate a direct role for childhood trauma in the occurrence of psychotic features when adjusting for more proximal risk factors and past history of psychotic disorder. A meta-analysis of studies of the effects of childhood adversity on persistent psychotic symptoms indicated high heterogeneity, complicating interpretation (34). Recent trauma was associated with psychotic features, and the reverse was also seen—the presence of psychotic features increased the vulnerability to subsequent traumatic events.

Mental disorders, including an important role for psychotic disorders, are increasingly appreciated as adding to mortality risk [35–37]. The present findings extend previous reports from the Hotel Study that in younger (<55 years old) participants, psychotic disorders contribute to mortality risk [23,34,38]. Psychotic disorders are additive with other risk factors observed here, including hepatic fibrosis and alcohol dependence, complementing findings related to HIV and HCV in another study from this neighborhood [51]. Comprehensive, integrated interventions are needed to address the trimorbidity of substance use and mental and physical illnesses that burden socially marginalized people worldwide [1–5].

Among the strengths of the study are the relatively large numbers of participants from a community-based sample with frequent longitudinal follow-up assessments. There are also limitations. The community sample was recruited in a Canadian neighborhood where nonprescribed substance use is widespread and universal healthcare is available. While the demographics of study participants were similar to those in other studies carried out in this and similar neighborhoods across Canada [65–68], in other contexts, drivers for psychosis, including migration, may differ and affect generalizability. Since the likelihood of psychosis was similar during periods of precarious housing or homelessness, these results may also apply to people at risk of homelessness. Similar to other longitudinal studies, missed visits may affect precision of the results, and unidentified factors may contribute to missingness, though no evidence of systematic bias was identified on sensitivity or multiple imputation analyses. The analyses here were not designed to assess the possible differences in patterns of psychotic features between participants who were substance users compared with substance dependence, substance-induced psychotic disorders, or other forms of psychotic disorders [69,70]. The focus was on the occurrence of psychotic features; the likelihood of transition or progression from one form of psychotic disorder to another over a 1-year or longer period deserves additional attention [6,7,71,72]. As well, our assessment approach focused on identifying the presence of psychotic features in a heterogeneous, community-based sample of participants living in precarious housing or homelessness. The same symptom severity thresholds were applied in other studies to identifying relapse from being asymptomatic to having recurrence of psychosis requiring clinical intervention [9,10]. This approach differs from severity thresholds used to assess improvement in treatment responsive patients with schizophrenia or in defining treatment-resistant schizophrenia [73,74]. A final limitation was the lack of useful family history information. One-third of the study participants grew up in foster care or with adoptive parents, and others experienced considerable adversity during childhood, limiting the feasibility of obtaining the necessary details.

In summary, this study of a community-based sample of people living in precarious housing or homelessness demonstrated multiple risk factors for psychotic features. A history of psychotic disorder increased the risk of early mortality in people under the age of 55 years. Ongoing substance use including methamphetamine, alcohol, and cannabis are modifiable risk factors that could be a focus for intervention.

## Supporting information

S1 STROBE Checklist (PDF)

S1 Table Demographics in studies of precariously housed and homeless participants in Vancouver, Canada. (PDF)

S2 Table Demographics in cross-Canada studies of precariously housed and homeless participants. (PDF)

S3 Table Descriptive values of functional measures at baseline. (PDF)

S4 Table Relationships between past psychotic disorder diagnosis, baseline psychotic features, and level of function at baseline. (PDF)

S5 Table Lagged effects of time-varying risk factors (the week prior to psychosis assessment week) on odds of observing psychotic features during the first year after entering the study. (PDF)

S6 Table Testing for reverse association or a relationship between psychotic features at an assessment visit and subsequent substance use (in the following week, measured in days) or any subsequent traumatic events (in the following month). (PDF)

S7 Table Effects of risk factors on odds of observing psychotic features during the first year after study entry by multiple imputation approach. (PDF)

S8 Table Screening assessment of risk factors for early mortality with unadjusted Cox proportional hazards regression analyses. (PDF)

S1 Protocol (PDF)

## Abbreviations

aHR: adjusted hazard ratio
AIC: Akaike Information Criterion
aOR: adjusted odds ratio
APRI: aspartate aminotransferase-to-platelet ratio index
CI: confidence interval
CT: computed tomography
DSM-IV-TR: Diagnostic and Statistical Manual for Mental Disorders-TR Fourth Edition
HCV: hepatitis C virus
HIV: human immunodeficiency virus
MRI: magnetic resonance imaging
OR: odds ratio
PANSS: Positive and Negative Syndrome Scale
qPCR: qualitative polymerase chain reaction
RFS: Role Functioning Scale
rTHQ: recent THQ
SE: standard error
SOFAS: Social and Occupational Functioning Assessment Scale
SRO: single-room occupancy
TBI: traumatic brain injury
THC: tetrahydrocannabinol
THQ: Trauma History Questionnaire
