# Supplementary material for: Associations of substance use, psychosis, and mortality among people living in precarious housing or homelessness: A longitudinal, community-based study in Vancouver, Canada
Source: PLoS Med. 2020 Jul 6;17(7):e1003172. doi: 10.1371/journal.pmed.1003172 (PMC7337288; doi:10.1371/journal.pmed.1003172)
Supplement: S1 STROBE Checklist — (PDF) [file pmed.1003172.s001.pdf]

STROBE Statement—Checklist of items that should be included in reports of ***cohort studies***

	Item No	Recommendation	Page No
**Title and abstract**	1	(*a*) Indicate the study's design with a commonly used term in the title or the abstract	Title
		(*b*) Provide in the abstract an informative and balanced summary of what was done and what was found	Abstract
**Introduction**			
Background/rationale	2	Explain the scientific background and rationale for the investigation being reported	Introduction paragraphs 1-3
Objectives	3	State specific objectives, including any prespecified hypotheses	Introduction paragraph 4
**Methods**			
Study design	4	Present key elements of study design early in the paper	Methods, paragraph 1
Setting	5	Describe the setting, locations, and relevant dates, including periods of recruitment, exposure, follow-up, and data collection	Methods, paragraph 1
Participants	6	(*a*) Give the eligibility criteria, and the sources and methods of selection of participants. Describe methods of follow-up	Methods, paragraph 1; Figure 1
		(*b*) For matched studies, give matching criteria and number of exposed and unexposed	n/a
Variables	7	Clearly define all outcomes, exposures, predictors, potential confounders, and effect modifiers. Give diagnostic criteria, if applicable	Methods, paragraphs 3-9
Data sources/ measurement	8*	For each variable of interest, give sources of data and details of methods of assessment (measurement). Describe comparability of assessment methods if there is more than one group	Methods, paragraphs 3-9
Bias	9	Describe any efforts to address potential sources of bias	Methods, paragraph 1;S1, S2 Tables
Study size	10	Explain how the study size was arrived at	Figure 1

Quantitative variables	11	Explain how quantitative variables were handled in the analyses. If applicable, describe which groupings were chosen and why	Statistical analysis paragraph 1
Statistical methods	12	(*a*) Describe all statistical methods, including those used to control for confounding	Statistical analysis
		(*b*) Describe any methods used to examine subgroups and interactions	Paragraph 4
		(*c*) Explain how missing data were addressed	Paragraph 3
		(*d*) If applicable, explain how loss to follow-up was addressed	Paragraph 3
		(*e*) Describe any sensitivity analyses	Paragraph 3
**Results**			
Participants	13*	(a) Report numbers of individuals at each stage of study—eg numbers potentially eligible, examined for eligibility, confirmed eligible, included in the study, completing follow-up, and analysed	Figure 1
		(b) Give reasons for non-participation at each stage	Figure 1
		(c) Consider use of a flow diagram	Figure 1
Descriptive data	14*	(a) Give characteristics of study participants (eg demographic, clinical, social) and information on exposures and potential confounders	Results paragraphs 1-2;Table 1
		(b) Indicate number of participants with missing data for each variable of interest	Figure 1; Tables 1-4
		(c) Summarise follow-up time (eg, average and total amount)	Results paragraphs 2 and 10
Outcome data	15*	Report numbers of outcome events or summary measures over time	Results paragraphs 2, 3, 10, 12

Main results	16	(*a*) Give unadjusted estimates and, if applicable, confounder-adjusted estimates and their precision (eg, 95% confidence interval). Make clear which confounders were adjusted for and why they were included	Results, Table 3, Fig 2, paragraph 6
		(*b*) Report category boundaries when continuous variables were categorized	n/a
		(*c*) If relevant, consider translating estimates of relative risk into absolute risk for a meaningful time period	n/a

Other analyses	17	Report other analyses done—eg analyses of subgroups and interactions, and sensitivity analyses	Results, paragraph 7
**Discussion**			
Key results	18	Summarise key results with reference to study objectives	Discussion, paragraph 1
Limitations	19	Discuss limitations of the study, taking into account sources of potential bias or imprecision. Discuss both direction and magnitude of any potential bias	Discussion, paragraph 8
Interpretation	20	Give a cautious overall interpretation of results considering objectives, limitations, multiplicity of analyses, results from similar studies, and other relevant evidence	Discussion, paragraphs 2-7
Generalisability	21	Discuss the generalisability (external validity) of the study results	Discussion, paragraph 8
**Other information**			
Funding	22	Give the source of funding and the role of the funders for the present study and, if applicable, for the original study on which the present article is based	Submission form

*Give information separately for exposed and unexposed groups.

Note: An Explanation and Elaboration article discusses each checklist item and gives methodological background and published examples of transparent reporting. The STROBE checklist is best used in conjunction with this article (freely available on the Web sites of PLoS Medicine at http://www.plosmedicine.org/, Annals of Internal Medicine at http://www.annals.org/, and Epidemiology at http://www.epidem.com/). Information on the STROBE Initiative is available at http://www.strobe-statement.org.
