# Supplementary material for: Associations of substance use, psychosis, and mortality among people living in precarious housing or homelessness: A longitudinal, community-based study in Vancouver, Canada
Source: PLoS Med. 2020 Jul 6;17(7):e1003172. doi: 10.1371/journal.pmed.1003172 (PMC7337288; doi:10.1371/journal.pmed.1003172)
Supplement: S1 Table — (PDF) [file pmed.1003172.s002.pdf]

S1 Table. Demographics in studies of precariously housed and homeless participants in Vancouver, Canada.

Measure	Hotel study participants at baseline N=437	Lewis et al. [1] N=628	Shannon et al. [2] N=1813	Palepu et al. [3] N=396
Sampling	Community-based (all consenting tenants) at SROs and Downtown Community Court	Stratified random sample of SROs	Facility-based at SROs	Randomly selected participants from 10 SROs or rooming houses (due to difficulties with access, modified to include meal programs, community health centres, drop-in centres); for homeless, randomly selected shelters and meal programs
Age (years)	40.6 (mean) SD 11.2	46 (mean)	42 (median)	42.0 (mean) SD 10.2
Sex (Male)	340/437 (78%)	496/628 (79%)	1375/1800 (76%)	244/393 (62%)
White	261/437 (60%)	426/628 (68%)		222/383 (58%)
Indigenous	113/437 (26%)	105/628 (17%)	498/1813 (28%)*	105/383 (27%)
Mixed/Other	63/437 (14%)	97/628 (15%)		56/383 (15%)
Average monthly income, CAD	$875 (median) n=425	$1109 (mean)		$1074 (median)
Previous homelessness	323/431 (75%)	327/628 (52%)	512/1812 (28%) †	
**Education**				
0-8 years	52/432 (12%)	69/628 (11%)		
8-11 years	145/432 (55%)	276/628 (44%)		178/391 (46%)
12 years	103/432 (24%)	138/628 (22%)		99/391 (25%)
>12 years	132/432 (31%)	138/628 (22%)		114/391 (29%)
Active Smoker	2394/414 (95%)	481/628 (77%)		
Injection drug use in past 1-6 months	256/426 (60%)		718/1813 (40%)	
Current alcohol dependence or problematic use	81/437 (19%)	126/628 (20%)		60/396 (15%)
HIV status	65/409 (16%)		462/1813 (26%)	
Previous HCV infection	257/409 (63%)		868/1813 (48%)	

SRO: single room occupancy hotel, SD: standard deviation, CAD: Canadian dollars,

*Indigenous versus non-Indigenous only reported
†Past 6 months only

1. Lewis M, Boyes K, McClanaghan D, Copas J. Downtown Eastside Demographic Study of SRO and Social Housing Tenants. City of Vancouver, Vancouver, 2008, pp 1-102.

2. Shannon K, Ishida T, Lai C, Tyndall MW. The impact of unregulated single room occupancy hotels on the health status of illicit drug users in Vancouver. Int J Drug Policy 2006;17:107-114.

3. Palepu A, Gadermann A, Hubley AM et al. Substance use and access to health care and addiction treatment among homeless and vulnerably housed persons in three Canadian cities. PLoS One 2013;8(10);e75133.
