# Supplementary material for: Associations of substance use, psychosis, and mortality among people living in precarious housing or homelessness: A longitudinal, community-based study in Vancouver, Canada
Source: PLoS Med. 2020 Jul 6;17(7):e1003172. doi: 10.1371/journal.pmed.1003172 (PMC7337288; doi:10.1371/journal.pmed.1003172)
Supplement: S2 Table — (PDF) [file pmed.1003172.s003.pdf]

S2 Table. Demographics in cross-Canada studies of precariously housed and homeless participants.

**Measure**	**Hotel study participants at baseline**	**Health and Housing in Transition Study [1,2]**			
	**N=437**	**Vancouver, Toronto, Ottawa N=1192**	**Vancouver N=396**	**Toronto N=399**	**Ottawa N=396**
Sampling	Community-based (all consenting tenants) at SROs and Downtown Community Court	Randomly selected participants from 10 SROs or rooming houses (due to difficulties with access, modified to include meal programs, community health centres, drop-in centres); for homeless, randomly selected shelters and meal programs			
Age (years)	40.6 (mean) SD 11.2	(values presented as groups rather than mean)	42.0 (mean) SD 10.2	43.5 (mean) SD 9.9	41.1 (mean) SD 11.4
Sex (Male)	340/437 (78%)	781/1188 (66%)	244/393 (62%)	258/399 (65%)	278/395 (70%)
White	261/437 (60%)	722/1156 (62%)	222/383 (58%)	203/379 (54%)	297/393 (76%)
Indigenous	113/437 (26%)	205/1156 (18%)	105/383 (27%)	52/379 (14%)	47/393 (12%)
Mixed/Other	63/437 (14%)	229/1156 (20%)	56/383 (15%)	124/379 (33%)	49/393 (12%)
**Education**					
<12 years	197/432 (46%)	529/1183 (45%)	178/391 (46%)	170/397 (43%)	181/394 (46%)
12 years	103/432 (24%)	277/1183 (23.4%)	99/391 (25%)	90/397 (23%)	87/394 (22%)
>12 years	132/432 (31%)	377/1183 (32%)	114/391 (29%)	137/397 (34%)	126/394 (32%)
Monthly Income, median CAD	$875, n=425	$900	$1074	$770	$825

1. Hwang SW, Aubry T, Palepu A et al. The health and housing in transition study: a longitudinal study of the health of homeless and vulnerably housed adults in three Canadian cities. Int J Public Health 2011;56:609–623.
2. Palepu A, Gadermann A, Hubley AM et al. Substance use and access to health care and addiction treatment among homeless and vulnerably housed persons in three Canadian cities. PLOS One 2013;8(10);e75133.
