# Supplementary material for: Associations of substance use, psychosis, and mortality among people living in precarious housing or homelessness: A longitudinal, community-based study in Vancouver, Canada
Source: PLoS Med. 2020 Jul 6;17(7):e1003172. doi: 10.1371/journal.pmed.1003172 (PMC7337288; doi:10.1371/journal.pmed.1003172)
Supplement: S3 Table — (PDF) [file pmed.1003172.s004.pdf]

S3 Table. Descriptive values of functional measures at baseline. Mean (SD).

	Past psychotic disorder diagnosis		Baseline psychotic features	
	Present n=259	Absent n=164	Present n=223	Absent n=200
**Social and Occupational Functioning Assessment Scale***	38.3 (9.9)	42.7 (11.1)	37.4 (9.7)	42.9 (10.8)
**Role Functioning Scale total score†**	11.5 (3.1)	12.6 (3.3)	11.2 (3.1)	12.8 (3.2)
**Role Functioning Scale Work Productivity**	1.7 (1.0)	2.0 (1.3)	1.7 (1.0)	2.0 (1.2)
**Role Functioning Scale Independent Living / Self Care**	3.2 (1.0)	3.5 (1.1)	3.2 (1.0)	3.6 (1.1)
**Role Functioning Scale Immediate Social Network Relationships**	3.5 (1.3)	3.8 (1.3)	3.3 (1.3)	4.0 (1.3)
**Role Functioning Scale Extended Social Network Relationships**	3.0 (0.9)	3.2 (0.9)	3.0 (0.9)	3.3 (0.9)

*The Social and Occupational Functioning Assessment Scale is rated 0-100 with higher scores representing better functioning.

†The Role Functioning Scale is comprised of four domains (work productivity, independent living, and immediate and extended social network relationships) each rated 1 to 7 with higher scores representing better function. Measures of function were assessed by a research assistant.
