# Supplementary material for: Associations of substance use, psychosis, and mortality among people living in precarious housing or homelessness: A longitudinal, community-based study in Vancouver, Canada
Source: PLoS Med. 2020 Jul 6;17(7):e1003172. doi: 10.1371/journal.pmed.1003172 (PMC7337288; doi:10.1371/journal.pmed.1003172)
Supplement: S4 Table — (PDF) [file pmed.1003172.s005.pdf]

S4 Table. Relationships between past psychotic disorder diagnosis, baseline psychotic features, and level of function at baseline (n=423).

	Model 1*		Model 2†		Model 3‡	
	**Estimate (SE)**	***p*-value**	**Estimate (SE)**	***p*-value**	**Estimate (SE)**	***p*-value**
**Social and Occupational Functioning Assessment Scale**						
Past psychotic disorder diagnosis	2.38 (0.53)	<0.001			1.39 (0.57)	0.02
Baseline psychotic features			2.84 (0.50)	<0.001	2.28 (0.55)	<0.001
**Role Functioning Scale total score**						
Past psychotic disorder diagnosis	0.65 (0.16)	<0.001			0.33 (0.17)	0.06
Baseline psychotic features			0.88 (0.15)	<0.001	0.75 (0.17)	<0.001
**Role Functioning Scale Work Productivity**						
Past psychotic disorder diagnosis	0.17 (0.06)	0.003			0.13 (0.06)	0.03
Baseline psychotic features			0.13 (0.05)	0.01	0.08 (0.06)	0.18
**Role Functioning Scale Independent Living / Self-Care**						
Past psychotic disorder diagnosis	0.17 (0.05)	0.002			0.09 (0.06)	0.12
Baseline psychotic features			0.23 (0.05)	<0.001	0.190 (0.06)	<0.001
**Role Functioning Scale Immediate Social Network Relationships**						
Past psychotic disorder diagnosis	0.18 (0.07)	0.006			0.04 (0.07)	0.58
Baseline psychotic features			0.35 (0.06)	<0.001	0.33 (0.07)	<0.001
**Role Functioning Scale Extended Social Network Relationships**						
Past psychotic disorder diagnosis	0.13 (0.04)	0.004			0.06 (0.05)	0.18
Baseline psychotic features			0.17 (0.04)	<0.001	0.15 (0.05)	0.002

*Model 1 estimates the relationship between past psychotic disorder diagnosis and each functional outcome at baseline, controlling for age and sex.
†Model 2 estimates the relationship between baseline psychotic features and each functional outcome at baseline, controlling for age and sex.

‡Model 3 includes the effects of both past psychotic disorder and baseline psychotic features, controlling for age and sex.
