# Supplementary material for: Associations of substance use, psychosis, and mortality among people living in precarious housing or homelessness: A longitudinal, community-based study in Vancouver, Canada
Source: PLoS Med. 2020 Jul 6;17(7):e1003172. doi: 10.1371/journal.pmed.1003172 (PMC7337288; doi:10.1371/journal.pmed.1003172)
Supplement: S5 Table — (PDF) [file pmed.1003172.s006.pdf]

S5 Table. Lagged effects of time-varying risk factors (the week prior to psychosis assessment week) on odds of observing psychotic features during the first year after entering the study.

	Unadjusted			Adjusted* (n=409, 3625 observations)	
	n, obs	OR (95% CI)	P-Value	OR (95% CI)	P-Value
**Covariates**					
Time (months)	428, 4294	0.94 (0.91-0.98)	<0.001	0.97 (0.93-1.01)	0.12
Age	428, 4294	0.96 (0.94-0.99)	0.002	1.00 (0.98-1.03)	0.86
Male	428, 4294	1.79 (0.94-3.39)	0.07	2.14 (1.19-3.87)	0.01
**Time-invariant risk factors**					
Past psychotic disorder diagnosis	428, 4294	14.05 (8.96-22.03)	<0.001	18.42 (10.96-30.98)	<0.001
THQ score by age 18†	420, 4207	1.18 (1.07-1.31)	0.001	—	—
Persistent sequelae of traumatic brain injury	428, 4294	1.36 (0.56-3.29)	0.49	—	—
Homelessness by age 18	424, 4256	1.16 (0.65-2.07)	0.62	—	—
**Time-varying risk factors over the first year after entering the study‡**					
**Prior week**					
Daily tobacco use	415, 3769	1.34 (0.81-2.18)	0.25	—	—
Days using alcohol	407, 3472	0.99 (0.91-1.08)	0.82	1.01 (0.93-1.09)	0.89
Days using methamphetamine	408, 3458	1.12 (1.03-1.22)	0.01	1.12 (1.03-1.22)	0.01
Days using cannabis	408, 3460	1.05 (0.99-1.11)	0.11	1.03 (0.98-1.09)	0.25
Days using cocaine	408, 3461	0.97 (0.92-1.03)	0.34	—	—
Days using opioid	408, 3460	1.03 (0.97-1.11)	0.32	—	—
**Concurrent month of psychosis assessment**					
rTHQ score	410, 3775	1.50 (1.16-1.93)	0.002	1.69 (1.27-2.23)	<0.001
Homelessness	428, 4230	1.35 (0.65-2.80)	0.41	—	—
Adequate antipsychotic treatment	427, 4158	2.78 (1.76-4.40)	<0.001	2.26 (1.34-3.80)	0.002

Adequate methadone therapy	427, 4147	0.90 (0.63-1.30)	0.58	—	—

Data are odds ratio (OR) and 95% confidence interval (CI). obs = observations, THQ = Trauma History Questionnaire, rTHQ = recent THQ.
*Adjusted model selected for optimal fit by AIC and likelihood ratio test. Fixed effects are adjusted for the included time-invariant, time-varying factors, and covariates. Random effects (standard deviation): Subject (1.97), Timepoint (0.23).
†Linear effects of THQ scores for number of traumatic events by age 18. Quadratic effects were not significant (p>0.10).
‡Linear effects of number of days of substance use in the week prior to psychosis assessment week, and rTHQ scores for the number of traumatic events (0, 1, or ≥2) in the month concurrent with assessment visit are reported. Quadratic effects were not significant (p>0.10).
