# Supplementary material for: Associations of substance use, psychosis, and mortality among people living in precarious housing or homelessness: A longitudinal, community-based study in Vancouver, Canada
Source: PLoS Med. 2020 Jul 6;17(7):e1003172. doi: 10.1371/journal.pmed.1003172 (PMC7337288; doi:10.1371/journal.pmed.1003172)
Supplement: S6 Table — (PDF) [file pmed.1003172.s007.pdf]

S6 Table. Testing for reverse association, or a relationship between psychotic features at an assessment visit, and subsequent substance use (in the following week, measured in days) or any subsequent traumatic events (in the following month).

	Alcohol use		Methamphetamine use		Cannabis Use		Cocaine Use		Any Traumatic Event*	
	**Estimate**	**SD**	**Estimate**	**SD**	**Estimate**	**SD**	**Estimate**	**SD**	**OR**	**95% CI**
Psychotic features without covariates	0.161	0.132	0.097	0.052	0.123	0.077	-0.039	0.076	1.27‡	1.04-1.55
Psychotic features with covariates†	0.088	0.117	0.064	0.052	0.103	0.078	-0.024	0.078	1.30‡	1.05-1.62

Standard deviation (SD), Odds ratio (OR), and 95% confidence interval (CI)
N=408, 3451 observations

*rTHQ score for the following month was dichotomized as experiencing any type of traumatic event. N=403, 3440 observations.
†Covariates included time, age, sex, past psychotic disorder diagnosis, days of other non-prescription substance use in the following week, and, in the following month, adequate antipsychotic treatment, and any type of traumatic event.
‡$p < 0.05$
