# Supplementary material for: Associations of substance use, psychosis, and mortality among people living in precarious housing or homelessness: A longitudinal, community-based study in Vancouver, Canada
Source: PLoS Med. 2020 Jul 6;17(7):e1003172. doi: 10.1371/journal.pmed.1003172 (PMC7337288; doi:10.1371/journal.pmed.1003172)
Supplement: S7 Table — (PDF) [file pmed.1003172.s008.pdf]

S7 Table. Effects of risk factors on odds of observing psychotic features during the first year after study entry by multiple imputation approach.

	**Adjusted* (n=437, 56,810 observations)**	
	**OR (95% CI)**	**P-Value**
**Covariates**		
Time (months)	0.97 (0.94-1.00)	0.03
Age	1.00 (0.99-1.02)	0.87
Male	1.47 (1.02-2.12)	0.04
**Time-invariant risk factors**		
Past psychotic disorder diagnosis	9.89 (7.17-13.63)	<0.001
THQ score by age 18	—	—
Persistent sequelae of traumatic brain injury	—	—
Homelessness by age 18	—	—
**Time-varying risk factors**		
**Concurrent week of psychosis assessment**		
Daily tobacco use	—	—
Days using alcohol	1.07 (1.00-1.14)	0.04
Days using methamphetamine	1.14 (1.07-1.22)	<0.001
Days using cannabis	1.08 (1.03-1.13)	0.003
Days using cocaine	—	—
Days using opioid	—	—
**Concurrent month of psychosis assessment**		
rTHQ score	1.58 (1.27-1.97)	<0.001
Homelessness	—	—
Adequate antipsychotic treatment	2.20 (1.55-3.13)	<0.001
Adequate methadone therapy	—	—

Data are odds ratio (OR) and 95% confidence interval (CI). THQ = Trauma History Questionnaire, rTHQ = recent THQ.
*Adjusted for all fixed and current risk factors, and covariates included.
