# Supplementary material for: Associations of substance use, psychosis, and mortality among people living in precarious housing or homelessness: A longitudinal, community-based study in Vancouver, Canada
Source: PLoS Med. 2020 Jul 6;17(7):e1003172. doi: 10.1371/journal.pmed.1003172 (PMC7337288; doi:10.1371/journal.pmed.1003172)
Supplement: S8 Table — (PDF) [file pmed.1003172.s009.pdf]

S8 Table. Screening assessment of risk factors for early mortality with unadjusted Cox proportional hazards regression analyses.

**Factor**	**n**	**Hazard ratio (95% CI)**	**Log-rank *p*-value**	**Schoenfeld *p*-value**
Psychotic features during first year after study entry	424	0.95 (0.60-1.50)	0.82	0.02
Age < 55 years	310	1.44 (0.74-2.80)	0.29	0.20
Age ≥ 55 years	114	0.60 (0.30-1.17)	0.13	0.53
Past history psychotic disorder	437	1.22 (0.77-1.93)	0.40	0.04
Age < 55 years	323	2.22 (1.05-4.71)	0.04	0.27
Age ≥ 55 years	114	0.76 (0.40-1.43)	0.39	0.97
APRI > 0.7	395	2.08 (1.26-3.44)	0.004*	0.61
HIV positive	409	2.06 (1.25-3.41)	0.005*	0.68
HCV seropositive	409	1.25 (0.71-2.19)	0.44	0.73
HCV qPCR positive	401	1.35 (0.83-2.20)	0.23	0.65
Daily cigarette smoking	437	0.86 (0.44-1.67)	0.65	0.37
Alcohol dependence past	436	1.36 (0.86-2.15)	0.19	0.31
Alcohol dependence baseline	437	2.14 (1.32-3.47)	0.002*	0.85
Cocaine dependence past	436	0.94 (0.52-1.70)	0.83	0.35
Cocaine dependence baseline	437	1.26 (0.72-2.22)	0.42	0.98
Methamphetamine dependence past	437	0.93 (0.55-1.56)	0.78	0.71
Methamphetamine dependence baseline	437	1.03(0.56-1.87)	0.93	0.34
Opioid dependence past	437	0.92 (0.58-1.47)	0.73	0.63
Opioid dependence baseline	436	0.90 (0.56-1.44)	0.66	0.13
Cannabis dependence past	437	0.84 (0.52-1.35)	0.46	0.49
Cannabis dependence baseline	437	0.77 (0.46-1.30)	0.33	0.22
