# Supplementary material for: Associations of substance use, psychosis, and mortality among people living in precarious housing or homelessness: A longitudinal, community-based study in Vancouver, Canada
Source: PLoS Med. 2020 Jul 6;17(7):e1003172. doi: 10.1371/journal.pmed.1003172 (PMC7337288; doi:10.1371/journal.pmed.1003172)
Supplement: S1 Protocol — (PDF) [file pmed.1003172.s010.pdf]

Hotel: Co-occurring psychosis, addiction and viral infection

Protocol

Summary

Background: Vancouver's Downtown Eastside (DTES) was profiled in a United Nations feature, which noted that in this neighbourhood, of about 10,000 people, the hepatitis C virus (HCV) infection rate is nearly 70%, and the HIV prevalence of 30% is comparable to Botswana. Many of those living in the DTES have serious and persistent mental illness, with high substance abuse comorbidity. Montaner notes that "In Canada, treatment is available for everyone who needs it-but you have to come and get it". This is not the case for many DTES residents.

Rationale and Objective: To improve our ability to prevent such a situation from developing elsewhere, and to intervene most effectively, we need to define the risk factors for living in this neighbourhood with these co-occurring illnesses. We need to know if these people are the severe and persistently mentally ill, who have drifted into poverty and increasingly severe substance abuse, or are they marginalized members of society caught up in an epidemic of stimulant abuse, with episodes of psychosis maintained by continued ingestion of cocaine and methamphetamine? **Our objective is to focus on those living in the single room occupancy (SRO) hotels in the neighbourhood or who are currently residing in Vancouver's Downtown Eastside and have been assigned a court date within the previous six months at the Downtown Community Court, (hereinafter collectively referred to as The Study Population) and determine how they came to be there, with these co-occurring illnesses. We further seek a clearer understanding of the capabilities of those afflicted with these complex illnesses, and to identify targets for intervention.**

Goals: The first goal is to investigate specific clinical features of substance abuse that provide a pathway to developing psychosis or contracting infectious disease. The second goal is to analyse good or poor trajectories of complex illness. We will investigate factors related to persistent psychosis, and factors, which may impair or enhance the ability to engage in and adhere to treatment.

Hypotheses
Risk Factors: In The Study Population, stimulant abuse is a risk factor for psychosis, injection drug use of opiates or stimulants is a risk factor for HIV and HCV infection, and other drug abuse (including tobacco, alcohol and cannabis) is not a specific risk factor for either outcome.
Persistence of psychosis:
1) Persistence of psychosis over a one year period of time is related to the type and pattern of drug use, the social environment (social network), and individual experience (depression and trauma).
2) Cognitive dysfunction, and abnormalities of brain structure at baseline, make persistent psychosis more likely. **Accessing and adhering with treatment:** Understanding the capabilities of people living with co-occurring illnesses through collecting data related to hypotheses 1 and 2 will reveal a mismatch between needs and existing services.

Specific Aims
1. We will recruit subjects from SRO Hotels in Vancouver (total n=530) and from the Downtown Community Court (n=70) for a total sample size of 600 participants. Each will have a detailed clinical and physical assessment related to risk factors for psychosis and infectious disease.
2. All will participate in a monthly follow-up for up to 20 years, to investigate factors, which contribute to persistent psychosis, and good or poor ability to access health care services and adherement to treatment.
3. Subjects will participate in studies to determine if cognitive dysfunction and subtle abnormalities of brain structure contribute to persistent psychosis, and good or poor ability to access health care services and adhere to treatment.

Significance: If the hypotheses concerning stimulant and injection drug use are supported, this will focus the attention of treatment efforts on these two aspects of addiction. Defining social networks as factors, which contribute to persistence or recovery from psychosis, and to the ability to engage and

adhere to treatment, will shift the attention of treatment from individual patients to strategies to engage members of social networks and communities. Understanding the potential associations with brain pathology and cognitive dysfunction will allow a realistic assessment of the capabilities of patients, and setting achievable goals for interventions.

Objectives

The first goal is to investigate specific clinical features of substance abuse that may provide a pathway to developing psychosis or contracting infectious disease. The second major goal is to analyse good or poor trajectories of complex illness – including addiction, psychosis, and infectious disease. We will investigate factors related to the adverse clinical outcomes of persistent psychosis, and factors, which may impair or enhance the ability to engage in and adhere to treatment of addictions, psychosis and infectious disease.

Hypotheses

Risk Factors

- In The Study Population, stimulant abuse is a risk factor for psychosis, injection drug use of opiates or stimulants is a risk factor for HIV and HCV infection, and other drug abuse (including tobacco, alcohol and cannabis) is not a specific risk factor for either outcome.

Persistence of psychosis

- Persistence of psychosis over a one year period of time is related to the type and pattern of drug use, the social environment (social network), and individual experience (depression and trauma).
- Cognitive dysfunction, and abnormalities of brain structure at baseline, make persistent psychosis more likely.

Accessing and adhering with treatment

- Understanding the capabilities of people living with co-occurring illnesses through collecting data related to hypotheses 1 and 2 will reveal a mismatch between needs and existing services.

Specific Aims

1. We will recruit subjects from SRO Hotels in the Vancouver Downtown Eastside and Downtown Community Court, requesting participation from all residents in each hotel (total n=530) or individuals residing in the Downtown Eastside and are attending court hearings at the Downtown Community Court (n=70) for a total sample size of 600 participants. Recruitment will continue until this number is reached. Longitudinal histories of drug use will be obtained, and current use documented with a urine drug screen. Psychosis will be assessed by obtaining medical records, cross sectionally by interview, and rated using symptom severity scales.
2. All subjects recruited in Aim 1 (n=600) will be followed with a single session interview on a monthly basis to determine if they are psychotic. Depression and experience of trauma as well as criminal and violent behaviour will also be recorded. Drug use over the previous 4 weeks will be assessed, and a urine drug screen obtained.
3. From the cohort of subjects described in Aim 1, we will recruit eligible subjects for more detailed assessment to include cognitive testing and an MRI scan.
4. We will collect a wide range of descriptive information concerning health services utilization at baseline and on a monthly basis. The relationship between changes in psychosis and physical health status and these contacts will be examined.

Plan of Investigation

Study design: overview

This is a naturalistic, longitudinal study with a baseline assessment, and monthly follow up visits for up to twenty years. Eligible subjects will participate in more detailed assessments involving cognitive testing and magnetic resonance imaging (MRI).

Inclusion criteria

All individuals living in any of the housing facilities managed by xxx or individuals who are currently residing in Vancouver's Downtown Eastside and have been assigned a court date at Downtown Community Court within the past 6 months will be invited to participate in this study.
Subjects must be English speaking.
In the event of study expansion, all participants will be re-consented with a revised consent form highlighting changes to the study protocol.
All new participants must meet the above criteria.

Exclusion criteria

Inability to give informed consent would result in exclusion. This could include the inability to communicate sufficiently in English to complete interviews and questionnaires. Capacity to provide informed consent will be assessed at baseline using the Informed Consent Checklist. At each follow-up visit, capacity to participate in the visit will be assessed using the Follow-up visit pre-screen. This assesses orientation, understanding of the study, willingness to participate, and knowledge of how to contact the research team. If in the interviewer's judgment, the subject is unlikely to provide reasonably valid information because of uncooperativeness or clinical mental state, the participant may be asked to return on another date. If necessary, subjects may be referred to one of the participating study psychiatrists for further assessment and clinical care. In an urgent situation the research assistant would accompany the subject to the Emergency Room.

Exclusion criteria for MRI and cognitive module
Those with a history of cranial, thoracic or abdominal surgery, with pacemakers, artificial joints or other metallic implants will be excluded from the MRI scan and cognitive module. Subjects that have agreed to participate in the MRI portion of the study will be pre-screened for any potential metal fragments in the body (particularly in the orbits) if they have had any history of doing metal work or have been involved in use/deployment of ammunitions/explosives, welding, piping etc.). In these cases an X-ray will be performed prior to the MRI scan.

Known illness or disability such as significant head injury, stroke or previous brain surgery which would interfere with cognitive testing.

Withdrawal from therapy or assessment

This is a naturalistic study and there is no experimental therapy as part of the investigation. Subjects are free to withdraw consent for assessments at any time during the study.

Timecourse and evaluations

Screening

All subjects will participate in the first 3 Modules of testing, administered over three sessions. Subjects with predominant stimulant use, and with predominant opiate use, will be offered the opportunity to participate in Module 4. All subjects will be offered the opportunity to be follow-up monthly.

Module 1: Diagnosis
This is completed in Session 1 by a trained research staff member.

Module 2: Symptoms and social function
This is completed in Session 2 by a trained research staff member.

Module 3: Physical Health
This is completed in Session 3. Subjects will be accompanied at the appointments by a trained research staff member.

Module 4: MRI scan and cognitive module
This module is for subjects who consent to additional assessments by research staff. In Session 3a, there is an interview concerning symptoms of psychosis, and an examination for movement disorders. This

will be carried out by a Psychiatry Clinical Research Fellow. In Session 3b, cognitive testing is carried out, by a research assistant and supervised by a Neuropsychologist. In Session 3c, subjects participate in a magnetic resonance imaging (MRI) scan Research staff will accompany subjects to the MRI Unit at UBC for Session 3c.

Interim assessments:

These occur on a monthly basis. All are carried out by research staff.

Outcome assessments:

These occur at months 6, 12, 18, 24, 30, 36, 42, 48, 54, 60, 66, 72, 78, 84, 90, 96, 102, 108, 114, 120, 126, 132, 138, 144, 150, 156, 162, 168, 174, 180, 186, 192, 198, 204, 210, 216, 222, 238, 234, and 240. All are carried out by research staff.

Clinician rated scales and data from records

Overview: instruments were chosen to allow collection of information directly related to the hypotheses of the study, and to feasibly assess subjects who may have quite severe psychiatric disorders in some cases.

1. Socio-Demographic Information: a standard interview incorporates questions used in surveys done by Statistics Canada (Canadian Community Health Survey). History of medical and psychiatric illness is incorporated in the Socio-demographic information, and will be supplemented by reviewing admission and discharge summaries from records of hospitalization to determine the psychiatric diagnosis at each admission. Records from doctors visits, and information held in secure, confidential databases accessed by co-investigators through the BC-Centre for Disease Control or the Centre for Excellence in HIV/AIDS will be reviewed similarly.

2. Initial Substance Use Interview: this brief series of questions concerns the age at first use of tobacco, alcohol, cannabis and other drugs.

3. Arizona Social Support Interview Schedule: this instrument is used to define social network membership, and the feelings about different types of interactions between network members (1).

4. Inventory of Socially Supportive Behaviors: this instrument measures the frequency of interactions between social network members, also referred to as "enacted support" (2).

5. Maudsley Addiction Profile: a self-report questionnaire used to measure drug use and high-risk behaviour in the past 30 days. Information redundant with the Timeline follow back (below) will be removed.

6.Timeline follow back: types, amounts and pattern of drug and tobacco use for the previous 4 weeks.

48. Timeline Follow Back Cannabis: types, amounts and patterns of cannabis use for the previous 4 weeks.

49. Medicinal Cannabis Questionnaire: a questionnaire used to explore the use of medicinal cannabis

7. Mini International Neuropsychiatric Interview: an abbreviated (25-30 min) semi-structured clinical interview used to collect information allowing a diagnosis of DSM-IV Axis I disorders.

8. International Personality Disorder Examination-Screener: A 5-10 minute self-report screening measure developed to screen for Axis II disorders in the *Diagnostic and Statistical Manual of Mental Disorders*, 4th edition (DSM-IV).

9. Positive and Negative Syndrome Scale (PANSS): this is a 30-item scale rated after an interview, used to assess the severity of a range of symptoms of psychosis. The full PANSS will be used only for subjects participating in Module 4 (which includes the cognitive testing and MRI scan). The full PANSS

will be carried out by a research psychiatrist or psychologist, with reliability established by testing on videotaped interviews.

Specific items of the PANSS (conceptual disorganization, unusual thought content, delusions, hallucinations and suspiciousness) will be rated for all subjects, at each visit, to establish the presence/absence of psychosis. These items overlap with BPRS items used in other studies, and can be assessed by research assistants. These approaches are reported to allow discrimination of patients with schizophrenia and substance abuse disorders from those with other psychiatric disorders comorbid with substance abuse (3).

10. Beck Depression Inventory (BDI): this is a self-report measure of depression. We plan to focus specifically on psychosis and depression in the present study. Alternative, more comprehensive assessments of symptoms would require more time for interviewing, and this could compromise the ability of subjects to participate and provide reliable information related to the primary goals of the project. The BDI scores are reported to discriminate between patients with mood disorders and substance abuse disorders from those with other psychiatric disorders comorbid with substance abuse (3).

11. Family Interview for Genetics Studies (FIGS): the screening questions from the FIGS will be used to document the presence of mental disorders and substance use in family members of subjects. This instrument was used in a study of the relatives of subjects with methamphetamine psychosis (4).

12. Social & Occupational Functioning Scale (SOFAS): a brief evaluation of overall function, rated by the research assistant.

13. Global Assessment of Functioning Scale (GAF): a brief evaluation of general symptoms and functioning, rated by the research assistant. Both the SOFAS and GAF are assessed to allow comparison with other studies.

14. Role Functioning Scale: a rating of daily functioning, with good psychometric properties (5).

15. Trauma History Questionnaire: this instrument measures exposure to traumatic life events and records frequency and age of exposure. We are interested in traumatic experiences at any time, including recent events and experiences which may occur during the longitudinal period of the study, not only childhood. We need to document the time and nature of the event with this instrument, while the diagnosis of posttraumatic stress disorder (if present) will come from the MINI. The THQ has been validated in persons with severe mental disorders (6).

16. Physical Health Screen (SF-36): this is a general screening instrument for physical and emotional health, and measures the extent to which poor health impairs function. The focus is on the previous four weeks. Normative values are available for the Canadian population (7).

17. Medication Use: prescription medication taken under the supervision of a family physician or specialist will be recorded. The method of obtaining the medications (pharmacy, daily dispensing pharmacy, other) will be determined. The PharmaNet database will also be consulted.

18. Time Line Follow Back (medical): similar to the approach used for substance use, but applied to adherence with medications prescribed for medical or psychiatric illness.

19. Health Services Questionnaire: a self-report questionnaire used to measure utilization of health resources and medical needs of subjects, designed to incorporate elements from the Canadian Community Health Survey.

20. Weight, height, body mass index: measured in order to assess physical health.

21. Extrapyramidal Symptoms Rating Scale (ESRS): this requires a short examination (10 minutes) to assess extrapyramidal symptoms and signs (8, 9). A video tape for training on this assessment will be provided.

22. Barnes Akathisia Rating Scale: subjects are observed while seated and standing, for 2 minutes each to make these ratings (fully overlaps with the exam for the ESRS above) (10). Direct questioning is also used.

23. Soft signs scale: neurological soft signs are recorded on the Cambridge Neurological Inventory (CNI). Three of these CNI subscales address soft signs (motor coordination, sensory integration, and disinhibition). The examination overlaps with the ESRS above (22).

47. Gauteng Neurocognitive Assessment:
This assessment is a standardized neural/behavioral measure for marginalized populations with lower literacy rates and fewer educational years. It is being implemented here in attempt to characterize neural/behavioral abnormalities. (58)

24. Medical Review Questionnaire: This measure asks questions regarding past and current medical history.

26. Conflict Tactics Scale: This scale measures aggressive behaviour, including aggression that the individual has engaged in towards others and aggressive behaviour that has been perpetrated against the individual. It allows for an examination of the frequency of aggressive behaviours, and the types of aggressive behaviours that occur. (28)

29. Sensation Seeking Scale – Form V: This is a 40-item, forced choice inventory to measure individual differences in stimulation and arousal needs. There are four interrelated subscales including boredom susceptibility, disinhibition, experience seeking and thrill and advanture seeking. (35, 36, 37, 38).

30. Barratt Impulsiveness Scale: Version 11: The Barratt Impulsiveness Scale, Version 11 (BIS-11) is a 30 item self-report questionnaire designed to assess general impulsiveness taking into account the multi-factorial nature of the construct (39, 40, 41, 42, 43, 44, 45).

41. The Resilence Scale for Adults: This scale will be used to assess social competence, family cohesion, planned future, structured style and perception of self. (51)

43. Housing Questionnaire: This questionnaire will help track participant movement in terms of housing and time spent either homeless or in jail on a monthly basis to gather uptodate information.

44. Childhood Adversity and Neglect (C.A.N) Questionnaire:
The Childhood Adversity and Neglect (CAN) questionnaire is a 75-item questionnaire that assesses for a number of domains related to childhood adversity, abuse, neglect, and enrichment. It uses Likert scales and asks participants to respond to how often they had various childhood experiences. The CAN covers experiences up to age 18. (57)

45. Personal Recovery Outcome Measure (PROM):
This 40 Question self-report will help illuminate an individuals "way of living a satisfying, hopeful, and contributing life even with the limitations caused by illness". The aim is to help develop a clinically meaningful conceptual and measurement model for personal recovery of people who experience serious mental illness. (56)

46. Brain Injury Screening Questionnaire (BISQ):
A 29-item questionnaire designed to help to identify and determine if an individual has experienced an injury to the brain at any time and what sort of particular problems this injury may have caused (55).

Laboratory tests

31. Urine drug screen: a drug detection test that will determine use in the prior 48-hrs of – amphetamines, methamphetamine, barbiturates, benzodiazepines, cocaine (crack), marijuana, methadone, MDMA (ecstasy), opiates, tricyclic antidepressants. This will provide important, objective information on the role of ongoing use of substances in persistent psychotic symptoms. Further, the

urine samples will be sent to LifeLabs for testing of opioid analogs found in the community which cannot be detected by commercial urine drug screens.

32. CBC and differential: a measure of general physical health.

33. Serum cytokines: research measures of inflammation, related to mental health and general physical health.

34. Serology for HIV, Hepatitis B and Hepatitis C, HSV and CMV: testing in subjects with no available results from medical records. Participants with positive Hepatitis C serology will also have a PCR test to determine if virus is present.

35. Screening for human viruses: this is a screening research test which tests for any of the approximate 200 human viruses using the ViroChip in Dr. Tang's laboratory at the BC Centre for Disease Control. If the screening test for other human viruses indicates infection with hantavirus, hemorrhagic fever viruses, encephalitis viruses, other hepatitis viruses, measles, mumps, polio, rubella, SARS, smallpox, West Nile virus, yellow fever, H5 influenza, or H7 influenza we are required by law to report this to the BC-CDC and we will do so. Follow-up investigations will be initiated if clinically or epidemiologically indicated in consultation with the medical microbiologists and epidemiologists at BCCDC.

36. Metabolic Measures: Non-fasting lipids including Total Cholesterol, LDL Cholesterol, HDL Cholesterol, Triglycerides, Glycosylated Hemoglobin (HbA1C) and Glucose. These measures complement the medical history and systems review. Liver function will be assessed with ALT and AST levels.

37. Neuropsychological tests
English language acculturation questionnaire: this short interview concerns when subjects first learned English.

Day of evaluation questionnaire: this short interview concerns medications and drug use in the past 48 hours prior to the cognitive testing.

Wechsler Test of Adult Reading (WTAR): This test provides an estimate of an individual's level of premorbid intellectual functioning (24). The WTAR is administered during the screening visit only.

Stroop Color and Word Test: Tests the ability of the individual to separate word and color naming stimuli. This requires sustained attention and inhibition of a dominant response set. Stimulant abusers have been shown to have increased response latencies suggestive of difficulty inhibiting information on the Stroop task (11).

Intradimensional-extradimensional (ID/ED) shift task: Attentional shifting to attributes of a complex stimulus array will be evaluated with the CANTAB ID/ED Shift Task (12, 13). This task shares many features with the Wisconsin Card Sorting Test, a test broadly considered to be a measure of frontal lobe functioning (14). The ID/ED shift task has been used in studies of stimulant abusers and in other types of drug abuse (15).

Rapid Visual Information Processing (RVIP) Task: This CANTAB test requires monitoring and responding to specific digit sequences and inhibiting responses to distracters (16, 17).

Trail Making Test: A timed connect-the-dots. Participants are required to connect the dots in either a 1-2-3 sequence, or a 1-A-2-B-3-C pattern. (52)

Word Fluency Task: An evaluation of lexical or categorical fluency. (53)

Symbol Digit Modalities: This test will measure the participant's ability to associate a set of symbols and digits. (54)

Hopkins Verbal Learning Test-Revised: this is a brief assessment of memory, which includes many of the elements also found in detailed tests such as the California Verbal Learning Test (18).

Iowa Gambling Task: Decision making in response to differential incentive conditions will be examined with the Gambling Task, which is sensitive to orbitofrontal functioning(19) and will be used to evaluate decision-making. Poor decision-making has been detected in substance dependent individuals (20), including MA abusers (15). Furthermore, dysfunction of the orbitofrontal cortex has been specifically implicated in MA abuse (21).

Handedness Questionnaire: A 10-item questionnaire to help determine right versus left hand dominance for several activities will be administered. Cerebral dominance is important account for in studies of brain structure and function. Consequently, the Edinburgh Handedness Inventory (Oldfield, 1971) will be administered as a measure cerebral dominance. This questionnaire generates laterality quotients, or handedness scores, ranging from 0 (completely left-handed) to 100 (completely right-handed) (23).

Magnetic resonance imaging
38. MRI pre-screen: this is a short interview to determine eligibility for the MRI scan.

39. Blood Pressure Measurement: Blood pressure measurement will take place at baseline and at two follow-up visits. The BP-Tru monitor will be used given its proven efficacy (30, 31). Measurements will be taken at 1 minute intervals (32). Measurements will be correlated with degree of white matter hyperintensities on MRI FLAIR images to assess the degree to which hypertension affects brain health and cognition in this population, given that the most supported etiological theory for the development of cerebral white matter hyperintensities is ischemia (33, 34). If severe hypertensive measurements are recorded (according to B.C. guidelines) (48); blood pressure more than or equal to 180/110 mmHg, and/or diastolic pressure more than or equal to 130 mmHg), and/or if concerning symptoms of severe headache, nausea, vomiting, or visual changes, the person will be referred to medical services immediately.

Compensation

Participants will receive $xx for each 1 hour interview in Modules 1-4, $xx for the second and subsequent neurocognitive assessments, and $xx for the MRI scan in Modules 4. Travel expenses will be covered by the study.

References

1. Barrera M: A method for the assessment of social support networks in community survey research. Connections 1980; 3:8-13
2. Barrera M, Sandler IN, Ranmsey TB: Preliminary development of a scale of social support: Studies on college students. Am J Comm Psychol 1981; 9:435-447
3. Lykke J, Hesse M, Austin SF, Oestrich I: Validity of the BPRS, the BDI and the BAI in dual diagnosis patients. Addictive Behaviors 2008; 33:292-300
4. Chen C-K, Lin S-K, Sham PC, Ball D, Loh E-W, Murray RM: Morbid risk for psychiatric disorder among the relatives of methamphetamine users with and without psychosis. Am J Med Genet 2005; 136B:87-91
5. Goodman SH, Sewell DR, Cooley EL, Leavitt N: Assessing levels of adaptive functioning: the Role Functioning Scale. Community Mental Health J 1993; 29:119-131
6. Mueser KT, Salyers MP, Rosenberg SD, Ford JD, Fox L, Carty P: Psychometric evaluation of trauma and posttraumatic stress disorder assessments in persons with severe mental illness. Psychol Assess 2001; 13:110-117
7. Hopman WM, Towheed T, Anastassiades T, Ternenhouse A, Poliquin S, Berger C, Joseph L, Brown JP, Murray TM, Adachi JD, Hanley DA, Papadimitropoulos E: Canadian normative data for the SF-36 health survey. CMAJ 2000; 163:265-271
8. Gharabawi GM, Bossie CA, Lasser RA, Turkoz I, Rodriguez S, Chouinard G: Abnormal involuntary movement scale (AIMS) and extrapyramidal symptom rating scale (ESRS): cross-scale comparison in assessing tardive dyskinesia. Schizophrenia Res 2005; 77:119-128
9. Chouinard G, Margolese HC: Manual for the Extrapyramidal Symptom rating Scale (ESRS). Schizophrenia Res 2005; 76:247-265
10. Barnes TRE: A rating scale for drug-induced akathisia. Brit J Psychiatry 1989; 154:672-676
11. Salo R, Nordahl TE, Possin K, Leamon M, Gibson DR, Galloway GP, Flynn NM, Henik A, Pfefferbaum A, Sullivan EM: Preliminary evidence of reduced cognitive function in methamphetamine-dependent individuals. Psychiatry Res 2002; 111:65-74
12. Owen AM, Roberts AC, Polkey CE, Sahakian BJ, Robbins TW: Extra-dimensional versus intra-dimensional set shifting following frontal lobe excisions, temporal lobe excisions or amygdalo-hippocampectomy in man. Neuropsychologia 1991; 29:993-1006
13. Downes JJ, Roberts AC, Sahakian BJ, Evenden JL, Morris RG, Robbins TW: Impaired extra-dimensional shift performance in medicated and unmedicated Parkinson's disease: evidence for a specific attentional dysfunction. Neuropsychologia 1989; 27:1329-1343
14. Sahakian BJ, Owen AM: Computerised assessment in neuropsychiatry using CANTAB. J Royal Soc Med 1992; 85:399-402
15. Ornstein TJ, Iddon JL, Baldacchino AM, Sahakian BJ, London M, Everitt BJ, Robbins TW: Profiles of cognitive dysfunction in chronic amphetamine and heroin abusers. Neuropsychopharmacol 2000; 23:113-126
16. Sahakian BJ, Jones GMM, Levy R, Gray JA, Warburton DM: The effects of nicotine on attention, information processing, and short-term memory in patients with dementia of the Alzheimer tpe. Birit J Psychiatry 1989; 154:797-800
17. Wesnes K, Warburton DM: Effects of scopolamine and nicotine on human rapid information processing performance. Psychopharmacol 1984; 82:147-150
18. Benedict RHB, Schretlen D, Groninger I, Brandt J: Hopkins Verbal Learning Test- Revised: normative data and analysis of inter-form and test-retest reliability. Clin Neuropsychologist 1998; 12:43-55
19. Bechara A, Tranel D, Damasio H: Characterization of the decision-making deficit of patients with ventromedial prefrontal cortex lesions. Brain 2000; 123:2189-2202
20. Bechara A, Damasio H: Decision-making and addiction: impaired activation of somatic states in substance dependent individuals when pondering decisions with negative future consequences. Neuropsychologia 2002; 40:1675-1689
21. Volkow ND, Chang L, Wang G-J, Fowler JS, Franceschi D, Sedler M, Gatley SJ, Miller EA, Hitzemann R, Ding Y-S, Logan J: Loss of dopamine transporters in methamphetamine abusers recovers with protracted abstinence. J Neurosci 2001; 21:9414-9418
22. Chen, YH, Shapleske, J, Luque, R, McKenna, PJ, Hodges, JR, Calloway, P, Hymas, NFS, Dening, TR, Berrios, GE: The 9echsler9 neurological inventory: A clinical instrument for assessment of soft neurological signs in psychiatric patients. Psychiatry Research 1995; 56:183-2

23. Oldfield, R.C. (1971). The assessment and analysis of handedness: The Edinburgh Inventory. Neuropsychologia, 9, 97-113.
24. Green, R. E. A., Melo, B., Christensen, B., Ngo, L., Monette, G., & Bradbury, C. (2008). Measuring premorbid IQ in traumatic brain injury: An examination of the validity of the 10echsler test of adult reading (WTAR). Journal of Clinical and Experimental Neuropsychology, 30(2), 1-10.
25. Lenzenweger, M. F., Lane, M. C., Loranger, A. W., & Kessler, R. C. (2007). DSM-IV personality disorders in the national comorbidity survey replication. Biological Psychiatry, 62(6), 553-564.
28. http://en.wikipedia.org/wiki/Conflict_Tactics_Scale - cite_ref-1Straus, Murray A., Sherry L. Hamby, Susan Boney-McCoy, and David B. Sugarman. "The Revised Conflict Tactics Scales (CTS2): Development and Preliminary Psychometric Data." *Journal of Family Issues* 17.3 (1996): 283-316.
30. Graves, J.W., Nash, C., Burger, K., Sheps, S.G. (2003). Clinical decision-making in hypertension using an automated (BpTRU™) measurement device. Journal of Human Hypertension, 17(12), 823-827.
31. Beckett, L., and Godwin, M. (2005). The BpTRU automatic blood pressure monitor compared to 24 hour ambulatory blood pressure monitoring in the assessment of blood pressure in patients with hypertension. BMC Cardiovascular Disorders, 5:18.
32. Myers, M.G., Valdevieso, M., and Kiss, A. (2008). Optimum frequency of office blood pressure measurement using an automated sphygmomoanometer. Blood Pressure Monitoring, 13(6):333-8.
33. Gouw, A.A., Seewann, A., van der Flier, W.M., Barkhof, F., Rozemuller, A.M., Scheltens, P., Geurts, J.J. (2011). Heterogeneity of small vessel disease: a systematic review of MRI and histopathology correlations. Journal of Neurology Neurosurgery and Psychiatry, 82(2):126-35.
34. Debetter, S., and Markus, H.S. (2010). The clinical importance of white matter hyperintensities on brain magnetic resonance imaging: a systematic review and meta-analysis. British Medical Journal, 341, c3666.
35. Zuckerman, M. (1979). Sensation seeking: Beyond the optimal level of arousal. Hillsdale, NJ: Erlbaum.
36. Zuckerman, M. (1994). Behavioral expressions and biosocial bases of sensation seeking. New York: Cambridge University Press.
37. Zuckerman, M. (2007). Sensation seeking and risky behavior. Washington, DC: American Psychological Association.
38. Roberti JW, Storch EA, Bravata E. (2003). Further psychometric support for the Sensation Seeking Scale – Form V. Journal of Personality Assessment. 81 (3): 291-292.
39. Barratt, E. S. (1985). Impulsiveness subtraits: Arousal and information processing. In J. T. Spence and C. E. Izard (Eds.), Motivation, Emotion and Personality (pp. 137-146). Elsevier Science, North Holland.
40. Barratt, E. S. (1959). Anxiety and impulsiveness related to psychomotor efficiency. Perceptual and Motor Skills, 9, 191-198.
41. Eysenck, S. B. G. & Eysenck, H. J. (1977). The place of impulsiveness in a dimensional system of personality description. British Journal of Social and Clinical Psychology, 16, 57-68.
42. Gerbing, D. W., Ahadi, S. A. & Patton, J. H. (1987). Toward a conceptualization of impulsivity: Components across the behavioral and self-report domains. Multivariate Behavioral Research, 22, 357-379.
43. Luengo, M. A., Carrillo-de-la-Pena, M. T. & Otero, J. M. (1991). The components of impulsiveness: A comparison of the I.7 impulsiveness questionnaire and the Barratt impulsiveness scale. Personality and Individual Differences, 12, 657-667.
44. Patton, J. H., Stanford, M. S. & Barratt, E. S. (1995). Factor structure of the Barratt impulsiveness scale. Journal of Clinical Psychology, 51, 768-774.
45. Twain, D. C. (1957). Factor analysis for particular aspects of behavioral control – impulsivity. Journal of Clinical Psychology, 13, 133-136.

48. http://www.bcguidelines.ca/guideline_hypertension.html#part1. Ministry of Health, Government of British Columbia. Hypertension – Detection, diagnosis and management, February, 2008.

51. Friborg, O., Hjemdal, O., Rosenvinge, J. H., Martinussen, M. (2003). A new rating scale for adult resilience: What are the central protective resources behind healthy adjustment? *International Journal of Methods in Psychiatric Research, 12*, 65-76.

52. Reitan, R. M., & Wolfson, D. (1985). The Halstead–Reitan Neuropsycholgical Test Battery: Therapy and clinical interpretation. Tucson, AZ: Neuropsychological Press.

53. Gladsjo JA, Schuman CC, Evans JD, Peavy GM, Miller SW, Heaton RK (1999). Norms for letter and category fluency: Demographic corrections for age, education, and ethnicity. *Assessment*. 6(2):147-78. [PMID: 10335019]

54. Smith, Aaron (1982). The Symbol Digit Modalities Test: Western Psychological Services

55. Gordon WA, Brown M, Hibbard M. *Brain Injury Screening Questionnaire (BISQ) guidelines for web based version*. New York (NY): Mount Sinai Medical Center; 2010

56. Barbic, S., Kidd, SA., McKenzie, K. Personal Recovery Outcome Measure. Publication in development phase.

57. Childhood adversity and psychosocial adjustment in old age. Wilson RS, Krueger KR, Arnold SE, Barnes LL, Mendes de Leon CF, Bienias JL, Bennett DA Journal: The American journal of geriatric psychiatry: official journal of the American Association for Geriatric Psychiatry 2006 Apr; 14(4) 307-15

58. Hurwitz T, Jonsson G (2014) The Gauteng Neurocognitive Assessment (G-NCA). University of Witwatersrand, Johannesburg, SA.

OVERVIEW CHART OF ASSESSMENTS

	Month		1	2	3	4	5	6, 18, 30, 42, 54, 66, 78, 90, 102, 114 126,138, 150,162, 174,186, 198,210, 222,234	7	8	9	10	11	12, 24, 36, 48, 60, 72, 84, 96, 108, 120 132,144, 156,168, 180,192, 204,216, 228,240
	**Session**	**1-4**	**5, 20, 35, 50, 65, 80, 95, 110, 125, 140 155 170 185 200 215 230 245 260 275 290**	**6, 21, 36, 51, 66, 81, 96, 111, 126, 141 156 171 186 201 216 231 246 261 276 291**	**7, 22, 37, 52, 67, 82, 97, 112, 127, 142 157 172 187 202 217 232 247 262 277 292**	**8, 23, 38, 53, 68, 83, 98, 113, 128, 143, 158 173 188 203 218 233 248 263 278 293**	**9, 24, 39, 54, 69, 84, 99, 114, 129, 144, 159 174 189 204 219 234 249 264 279 294**	**10-11, 25-26, 40-41, 55-56, 70-71, 85-86, 100-101, 115-116, 130-131, 145-146, 160-161 175-176 190-191 205-206 220-221 235-236 250-251 265-266 280-281 295-296**	**12, 27, 42, 57, 72, 87, 102, 117, 132, 147, 162 177 192 207 222 237 252 267 282 297**	**13, 28, 43, 58, 73, 88, 103, 118, 133, 148, 163 178 193 208 223 238 253 268 283 298**	**14, 29, 44, 59, 74, 89, 104, 119, 134, 149, 164 179 194 209 224 239 254 269 284 299**	**15, 30, 45, 60, 75, 90, 105, 120, 135, 150, 165 180 195 210 225 240 255 270 285 300**	**16, 31, 46, 61, 76, 91, 106, 121, 136, 151, 166 181 196 211 226 241 256 271 286 301**	**17-19, 32-34, 47-49, 62-64, 77-79, 92-94, 107-109, 122-124, 137-139, 152-154, 167-169 182-184 197-199 212-214 227-229 242-244 257-259 272-274 287-289 302-304**
	Module 1													
	Consent	X												
	Inclusion/Exclusion Criteria	X												
	Informed consent checklist	X												
	Follow-Up Visit Pre-Screen		X	X	X	X	X	X	X	X	X	X	X	X
1	Socio-Demographic Information	X												
2	Initial Substance Use Interview	X												
3	Arizona Social Support Interview Schedule	X						X						X
4	Inventory of Socially Supportive Behaviors	X						X						X
24	Medical Review Questionnaire	X												
5	Maudsley Addiction Profile	X	X	X	X	X	X	X	X	X	X	X	X	X
6	Time line follow back (street drugs and tobacco)	X	X	X	X	X	X	X	X	X	X	X	X	X
43	Housing Questionnaire		X	X	X	X	X	X	X	X	X	X	X	X
44	C.A.N.	X												
45	PROM	X						X						X
49	Medicinal Cannabis Questionnaire	X												
31	Urine drug screen	X	X	X	X	X	X	X	X	X	X	X	X	X

	Module 2													
7	Mini International Neuropsychiatric Interview	X												X
8	International Personality Disorder Examination – Screener (IPDE-Screener)	X												
9	PANSS key psychosis items	X	X	X	X	X	X	X	X	X	X	X	X	X
10	Beck Depression Inventory	X	X	X	X	X	X	X	X	X	X	X	X	X
11	FIGS screening questions	X												
12, 13	SOFAS / GAF	X						X						X
14	Role Functioning Scale	X						X						X
15	Trauma History Questionnaire	X						X						X
46	BISQ	X												

29	Sensation Seeking Scale – Form V (SSS-V)	X												X
30	Barratt Impulsiveness Scale Version 11 (BIS-11)	X												X
41	Resilience Scale for Adult	X												X
26	Conflict Tactics Scale	X						X						X
	Module 3													
16, 17	Physical health screen (SF-36)/medication use	X						X						X
18	Time line follow back (drugs taken with a prescription)	X	X	X	X	X	X	X	X	X	X	X	X	X
48	Time line follow back Cannabis	X	X	X	X	X	X	X	X	X	X	X	X	X
19	Health Services Questionnaire	X	X	X	X	X	X	X	X	X	X	X	X	X
20	Weight, height, body mass index	X												X
32	CBC and differential	X												X
33	Serum cytokines	X												X
34	Viral serology and viral testing as needed	X												X
35	Screening for human viruses (ViroChip)	X												X
36	Non-Fasting Cholesterol, liver function tests	X												X
	Module 4	**3a-c**												**18a-c**
9	Positive and negative syndrome scale (PANSS)	X												X
21	Extrapyramidal Symptom Rating Scale (ESRS)	X												X
22	Barnes Akathisia Scale	X												X
47	Gauteng Neurocognitive Assessment	X												X
23	CNI Soft Signs Scale	X												X
37	Cognitive testing (WTAR, Stroop, HVLT, Handedness, Acculuration, CANTAB, Trail Making, Word Fluency, Symbol Digit Modalities, Day of Evaluation, Gambling Task	X												X
38	Magnetic Resonance Imaging	X												X
39	Blood Pressure Measurement	X												X
